# Dopant-Induced Photoluminescence Emission in Zero-Dimensional Cesium Zinc Halide Phosphors

**DOI:** 10.1021/acsphyschemau.6c00102

**Published:** 2026-07-30

**Authors:** Sheikh Jobe, Lamia A. Siddig, Ahmed Elsir, Abbas Khaleel, Fathy Hassan, Nutifafa Y. Doumon

**Affiliations:** † Department of Chemistry, College of Science, 11239 United Arab Emirates University, Al Ain P.O. Box 15551, United Arab Emirates; ‡ Department of Materials Science and Engineering, 8082 The Pennsylvania State University, University Park, Pennsylvania 16802, United States; § Materials Research Institute and Department of Engineering Science and Mechanics, The Pennsylvania State University, University Park, Pennsylvania 16802, United States

**Keywords:** Zero-dimensional zinc halides, ion doping, self-trapped excitons, photoluminescence, tunable emission, optoelectronic materials, electronic structure, luminescence mechanisms

## Abstract

Zero-dimensional (0D) metal halides with isolated polyhedral structures have attracted attention in optoelectronic applications because of their distinctive optical properties, including strong light absorption, large Stokes shift, and tunable emission wavelength. These 0D metal halides exhibit wide structural diversity, such as Cu^+^, Sn^2+^, Zn^2+^, Mn^2+^, Sb^3+^, Ag^+^, and Ln^3+^-based metal halides. As a branch within the 0D metal halides, Zn metal halides exhibit numerous interesting characteristics, including simple synthesis, a wide bandgap, and stability of Zn^2+^ ions. The large separation between [ZnX_4_]^2–^ polyhedra provides space that minimizes interactions between polyhedral units and allows wavelength-dependent excitation, making Zn metal halides potential host materials that can accommodate dopant ions to induce a desired emission wavelength. As a newly emerging class of nonperovskite metal halides, it is necessary to provide a review article to develop a deeper understanding of the impact of ion doping on the optical, electronic, and spectroscopic properties of Zn metal halides. Elucidation of these properties is essential for tailoring emission wavelengths, optimizing luminescence performance, and elucidating photophysical mechanisms such as self-trapped excitons (STE), energy transfer, and defect states, thereby guiding the rational design of optoelectronics materials and devices.

## Graphical abstract

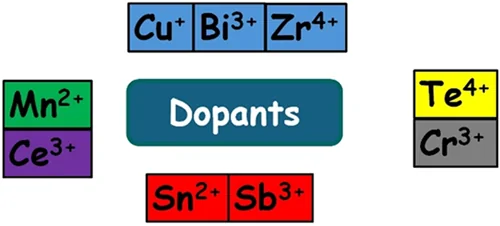

## 1 Introduction

Zero-dimensional (0D) metal halides are low-cost and solution-processable semiconductors that have captivated attention in optoelectronic applications such as light-emitting diodes (LEDs), photodetectors, and X-ray scintillators owing to their strong light absorption, tunable emission wavelength, large Stokes shift, and high photoluminescence quantum yield (PLQY).^1–3^ In a zero-dimensional electronic structure, excitons are confined in all three spatial dimensions.^4,5^ Moreover, in a 0D structure, the large separation between the polyhedra ensures that excitons are confined to individual polyhedral units, resulting in independent and highly efficient emissions. It is important to keep in mind that there is a wide variety of metal halides with remarkable differences in structural composition and dimensionality.^6–8^ The first pioneering work on cesium-based metal halides was reported in 2015, in which lead halide perovskites (CsPbX_3_) emerged as a novel luminescent material with high PLQY and a tunable emission wavelength across the visible light spectrum (400–700 nm).^9,10^ Thus far, extensive research has been conducted on CsPbX_3_ covering all aspects with regard to their optical, electronic, and morphological properties as well as their optoelectronic applications.^11,12^ Unfortunately, CsPbX_3_ contains metallic lead, which is hazardous to humans and requires special disposal procedures. To address the hazardous issue in CsPbX_3_, researchers have been looking into alternatives such as Sn^2+^, Mn^2+^, Cu^+^, Ag^+^, Zr^4+^, and Ln^3+^-based metal halides.^7,13^ Each of these metal halides exhibits its own distinct optical and electronic properties, which depend on the B-site cation that constitutes the octahedral or tetrahedral metal-halide structure.^6,7^ Sn^2+^, Mn^2+^, Cu^+^, Zr^4+^, and Ln^3+^ metal halides have been researched to a decent extent and their optical and electronic properties have also been reviewed.^5,7^ In comparison to 0D metal halides such as Cs_3_Cu_2_I_5_ or Cs_3_MnBr_5_, which exhibit high PLQY values (>80%), Zn metal halides are almost nonemissive, with low PLQY values of only 3.6%.^14–17^ In other words, Zn metal halides exhibit weak photoluminescence (PL) emission because Zn^2+^ has a 3d^10^ closed-shell electronic configuration that does not allow d-d transitions. In addition, the strong effective nuclear attraction of divalent Zn^2+^ stabilizes and lowers the energy of the 3d^10^ orbitals, thereby widening the energy gap between the filled 3d^10^ orbitals and the empty 4s orbital. The large energy gap between the filled 3d^10^ orbitals and the empty 4s orbital renders the 3d → 4s transition energetically unfavorable, making it the primary contributor to the weak PL emission, while other factors such as the parity-forbidden nature of the Zn^2+^ 3d → 4s electronic transition contribute to a lesser extent. That said, it is critical to highlight that Cu^+^ ions with a d^10^ electronic configuration also undergo parity-forbidden 3d → 4s transitions, yet Cu^+^-doped Zn metal halides (Cu^+^-doped Cs_3_ZnCl_5_) exhibit outstanding PLQY values of up to 90.2% owing to the smaller 3d-4s energy gap of Cu^+^ ions, resulting from their weaker effective nuclear attraction.^18,19^ This observation indicates that the 3d-4s energy gap, which is influenced by the effective nuclear attraction, has a more dominant impact on emission efficiency than the parity-forbidden nature of the transitions. The parity-forbidden nature of the transition primarily reduces the radiative transition probability, resulting in a prolonged radiative decay lifetime rather than intrinsically lowering the luminescence efficiency. In Cu^+^-doped Cs_2_ZnBr_4_, two parity-forbidden 3d → 4s transitions exist: one associated with Zn^2+^ ions and the other with Cu^+^ dopants. The introduction of Cu^+^ dopants provides an alternative luminescent pathway in which the Cu^+^ 3d → 4s transition, with its smaller energy gap, gives rise to intense blue PL emission.^16,20,21^ Nonetheless, the PL emission in Zn metal halides can be modulated by doping, which alters the electronic structure, introduces additional absorption bands, and generates new luminescent centers. Therefore, it is reasonable to propose that Zn metal halides serve as host materials capable of accommodating dopant ions, thereby inducing the desired emission wavelengths. Moreover, Zn metal halides are stable in air owing to their closed-shell 3d^10^ electronic configuration and therefore do not suffer from oxidation-related challenges that are observed in Sn (II) and Cu (I) metal halides.^22,23^ Metal halides overcome many of the limitations of conventional silicate host materials.^24–26^ In contrast to silicate host materials, which require high-temperature solid-state synthesis, typically above 1000 °C using specialized high-temperature furnaces, metal halides can be synthesized under much milder conditions, even at room temperature.^24–26^ In addition, metal halides exhibit higher absorption coefficients, more flexible crystal lattices that facilitate dopant incorporation, and greater spectral tunability through variation of the halide composition. The motivation behind this review article is to present Zn metal halides as an emerging class of nonperovskite metal halides. Pioneering work on Zn metal halides began around the year 2020, after which these materials were doped with various ions, including Ce^3+^, Sn^2+^, Mn^2+^, Cu^+^, Sb^3+^, Te^4+^, Eu^3+^, and Cr^3+^, as reported in the literature.^16,21^ For example, in 2020, Manna and coauthors doped Cu^+^ ions into Cs_2_ZnCl_4_ to induce a blue PL emission.^21^ Fast forward to 2025, Shi and coauthors doped Ce^3+^ ions into Cs_2_ZnBr_4_ to fabricate UV LEDs with exceptional anti-thermal quenching properties.^1^ Considering these achievements, it is essential to elucidate the impact of ion doping on the optical, electronic, and spectroscopic properties of Zn metal halides. A summary of review topics to be covered in this review article is presented in Figure 1.

**Figure 1:**
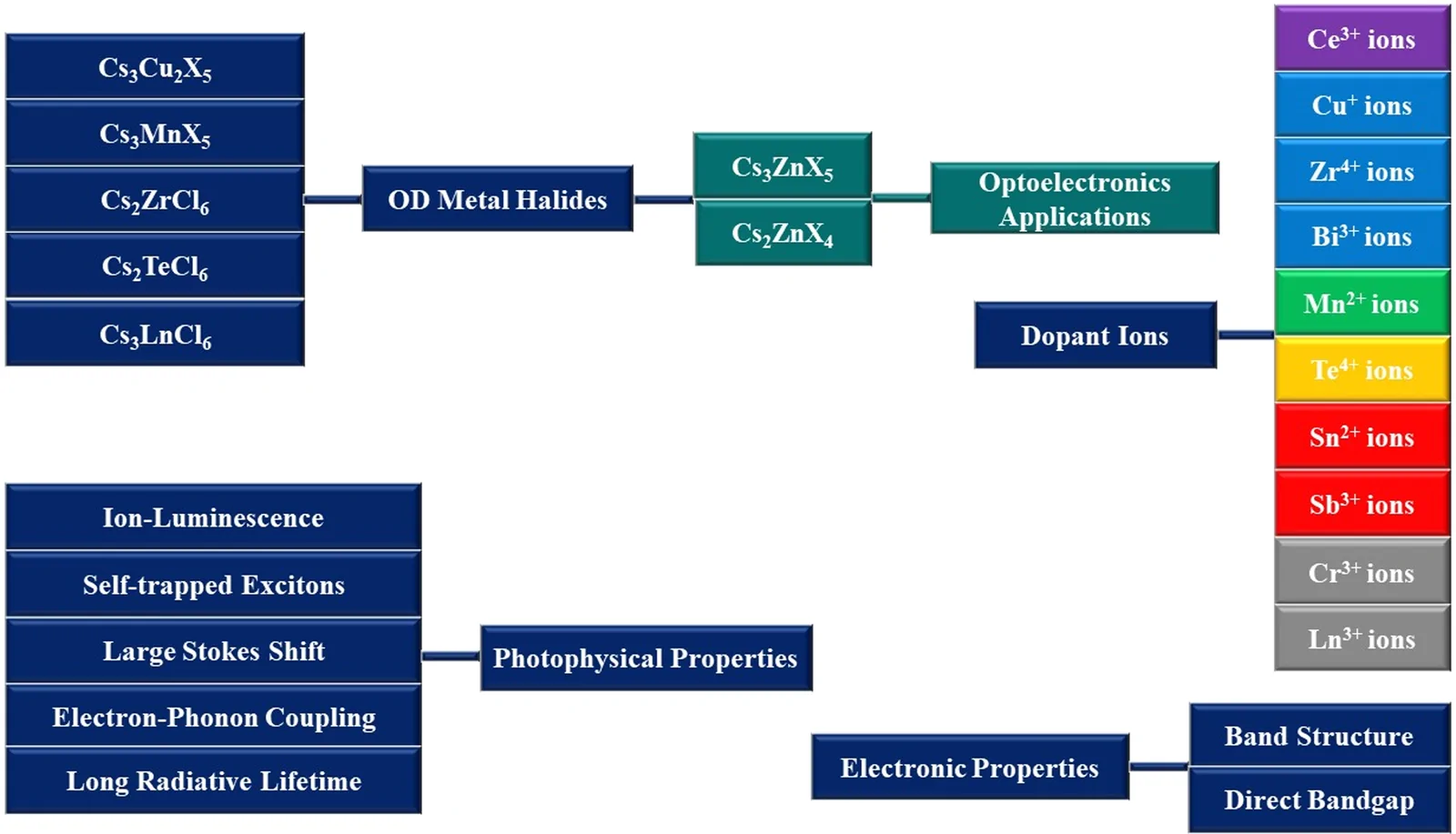
A summary of review topics.

## 2 Metal Halide Perovskites

In 2015, CsPbX_3_ emerged as a novel solution-processable semiconductor with high PLQY, broad absorption spectrum, high color purity, short radiative decay lifetime, and tunable emission wavelength across the visible light spectrum.^9,10,27^ In CsPbX_3_, Cs^+^ ions act as the A-site cation, Pb^2+^ occupies the B-site, and X^–^ represents the halide anion. In CsPbX_3_, the emission wavelength depends on the bandgap, which decreases as the halide composition changes from Cl to Br to I (Figure 2).^8,9^ This trend aligns with the experimental observation in which CsPbCl_3_ shows blue emission (410 nm), CsPbBr_3_ exhibits green emission (520 nm), and CsPbI_3_ presents red emission (650 nm), indicating a progressive red shift in PL emission due to a decreasing bandgap.^9,10^ In general, metal halide perovskites are classified as defect-tolerant because their defects are shallow, with energy levels situated above the conduction band or below the valence band, rather than deep states within the bandgap.^8,13^ Thus, these shallow defects do not trap charge carriers or act as nonradiative recombination centers, enabling the material to maintain strong optoelectronic performance despite the presence of lattice imperfections.^8,13^ Nonetheless, CsPbX_3_ is problematic, and current research trends are focused on exploring more sustainable and environmentally friendly materials.^7,13^ Researchers have replaced the B-site cation in CsPbX_3_ with alternatives such as Cu^+^, Mn^2+^, Zr^4+^, Sn^2+^, and Pt^4+^ ions.^28–31^ The size and valency of these cations determine the crystal structure of the metal halides. For instance, Cs_3_Cu_2_X_5_ and Cs_3_MnX_5_ form 0D structures, CsSnX_3_ forms a 3D perovskite structure, and Cs_2_PtCl_6_ and Cs_2_ZrCl_6_ adopt 0D double-perovskite structures.^28–31^

**Figure 2:**
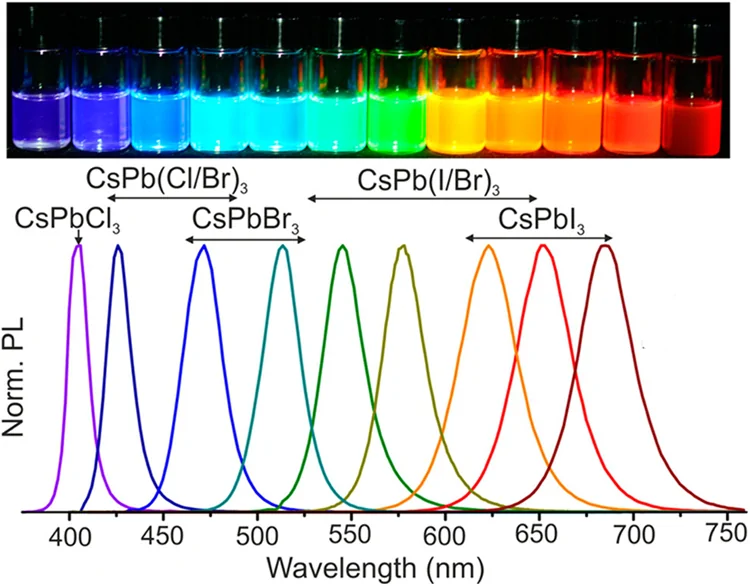
Tunable PL emission in CsPbX_3_ perovskites. Reproduced from ref 8. Copyright 2018, American Chemical Society.

### 2.1 0D Metal Halides

A zero-dimensional structure is a crystal system in which metal-halide polyhedra are isolated in all three spatial dimensions, resulting in isolated polyhedral units.^4,5^ In simple words, the polyhedral units do not share corners, edges, or faces with their neighbors.^4,5^ There is great diversity within 0D metal halides. 0D copper (I) halides, such as Cs_3_Cu_2_I_5_ and Cs_3_Cu_2_Br_5_, show blue emission, whereas Cs_3_Cu_2_Cl_5_ exhibits green emission.^15,32^ Likewise, 0D double perovskites such as Cs_2_ZrCl_6_ demonstrate blue emission, while Cs_2_TeCl_6_ presents yellow emission.^29,33^ In addition, Mn-based metal halides such as Cs_3_MnBr_5_, with tetrahedral coordination, exhibit green emission via Mn^2+^ d-d electronic transitions.^34,35^ Furthermore, Cs_3_LnCl_6_ metal halides show unique PL emission based on the B-site Ln^3+^ ions.^5,36^ Overall, these metal halides demonstrate a diverse and tunable PL emission as summarized in Table 1.

**Table 1:** PL Emission Wavelengths of 0D Metal Halides

B-site cation	compound	emission wavelength	PLQY	color	references
Cu^+^	Cs_3_Cu_2_I_5_	445 nm	49.2%	blue	^37^
Cu^+^	Cs_3_Cu_2_Br_5_	461 nm	17.3%	blue	^37^
Cu^+^	Cs_3_Cu_2_Cl_5_	515 nm	91.3%	green	^37^
Zr^4+^	Cs_2_ZrCl_6_	450 nm	38%	blue	^38^
Te^4+^	Cs_2_TeCl_6_	590 nm	6.7%	yellow	^33^
Mn^2+^	Cs_3_MnX_5_	520 nm	82%	green	^17^
Ln^3+^	Cs_3_LnCl_6_	Depends on B-site ions			^5^

### 2.2 Zinc Metal Halides

Zn metal halides exhibit a 0D crystal structure with chemical formulas of Cs_3_ZnX_5_ and Cs_2_ZnX_4_.^16,18^ The Zn^2+^ ions are in a tetrahedral geometry coordinated with four halide ions to constitute [ZnX_4_]^2–^ tetrahedra that are spatially isolated by large Cs^+^ cations, forming a 0D structure that crystallizes in either the orthorhombic *Pnma* or tetragonal I*4*/*mcm* space group.^16,18^ This 0D crystal structure indicates that the large separation between [ZnX_4_]^2–^ tetrahedra in Zn metal halides provides sufficient space to minimize interactions between the [ZnX_4_]^2–^ tetrahedra, thereby allowing wavelength-dependent excitation. Figure 3 lists all the dopant ions to be discussed in this review article. Although dopant ions such as Pb^2+^, Sn^2+^, Ce^3+^, and Zr^4+^ are known to adopt octahedral coordination in A_2_BX_6_ or related stoichiometries, numerous reports have shown that they can also occupy tetrahedral sites in Zn metal halides.^22,39,40^

**Figure 3:**
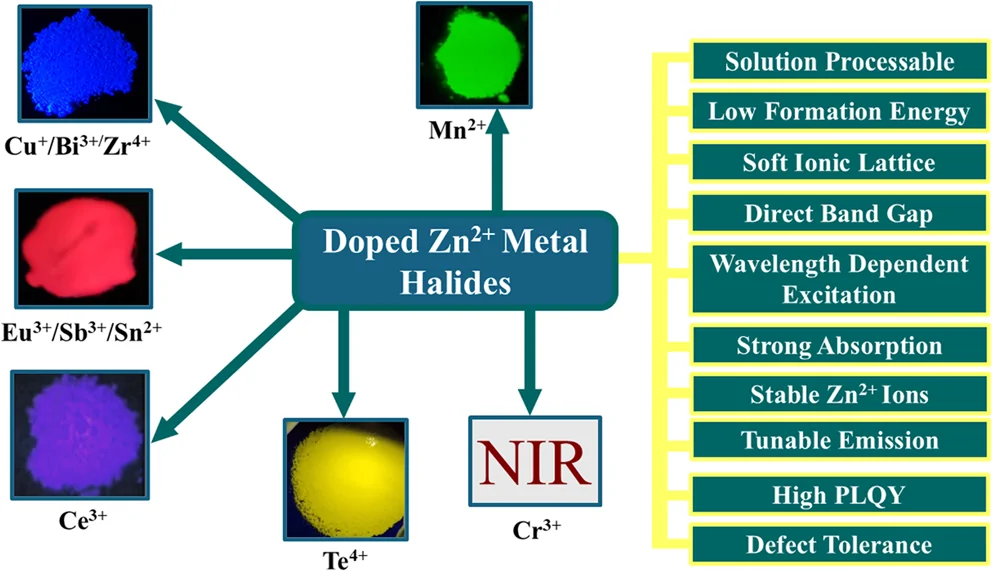
Dopant ions and their corresponding PL emission wavelengths.

The effect of doping on XRD patterns and crystal structures can be classified into three main categories based on the relative ionic radius of the dopant compared to Zn^2+^ ions. First, when the dopant ions are smaller than Zn^2+^ ions, lattice contraction occurs.^41^ This contraction causes X-ray diffraction (XRD) patterns to shift toward higher angles.^41^ Second, when the dopant ions have an ionic radius comparable to that of Zn^2+^ ions, minimal structural distortion is expected. In this case, there is little to no shift in XRD diffraction patterns, indicating that the lattice remains largely unchanged (Figure 4a,b).^20,23^ Lastly, when the dopant ions are larger than Zn^2+^, lattice expansion occurs.^1,22^ This expansion causes XRD diffraction patterns to shift toward lower angles. Overall, successful doping can be confirmed using simple characterization techniques such as PL spectroscopy, XRD, energy-dispersive X-ray spectroscopy (EDS) elemental mapping, and X-ray photoelectron spectroscopy (XPS) (Figure 4c,d).^20,23^

**Figure 4:**
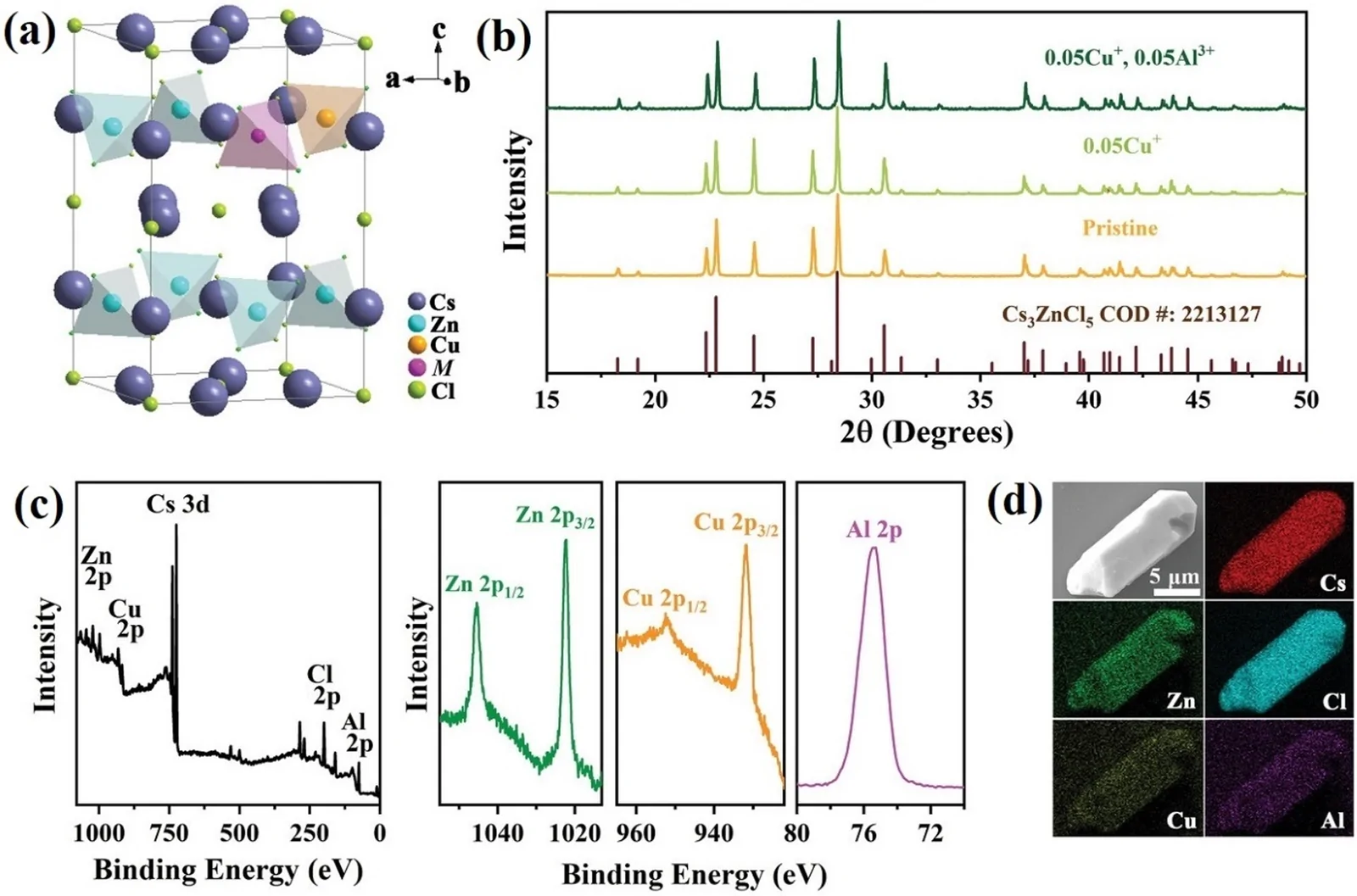
(a) Al^3+^ and Cu^+^ ions replacing Zn^2+^ in Cs_3_ZnCl_5_. (b) XRD patterns of doped and undoped Cs_3_ZnCl_5_. (c) XPS spectra of doped Cs_3_ZnCl_5_. (d) EDS mapping of doped Cs_3_ZnCl_5_. Reproduced from ref 23. Copyright 2022, Wiley.

## 3 Optical Properties and Emission Mechanisms

The optical properties and emission mechanism of metal halides are predominantly dictated by their electron configuration. This section discusses and provides insights into the three most common emission mechanisms in metal halides, namely free-exciton emission in lead halide perovskites, self-trapped exciton (STE) emission in 0D metal halides, and ion luminescence exhibited by trivalent lanthanide ions with partially filled f orbitals or transition-metal ions (Mn^2+^) with partially filled d orbitals.

### 3.1 Free Exciton Emission

An exciton is a bound pair of an electron and a hole that forms when a photon excites an electron from the valence band to the conduction band, creating a positively charged hole.^18^ Excitons are classified into two types, namely free excitons and bound excitons.^42^ A free exciton is defined as a delocalized electron–hole pair that is not trapped by defects, impurities, or lattice distortions, meaning it is free to move or propagate through the crystal as a quasiparticle.^13,42^ Overall, the free exciton formation process occurs in three steps (Figure 5a,b).^43^

**Figure 5:**
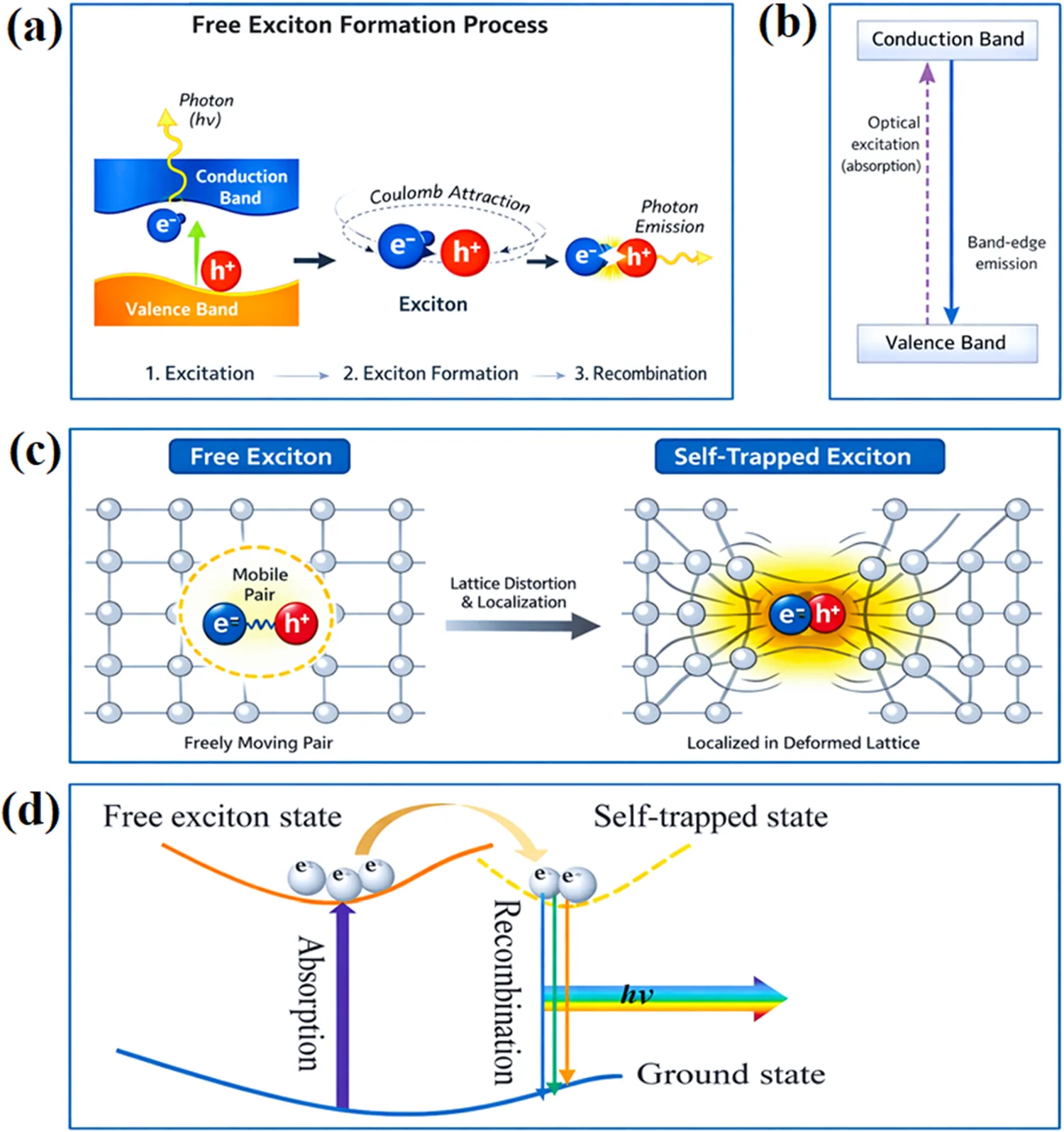
(a) Free exciton formation. (b) Band-edge emission mechanism in CsPbX_3_ or CsSnX_3_ perovskites. Reproduced from ref 43. Available under a CC-BY 4.0 license. Copyright 2024, Wiley. (c) STE lattice distortion and localization. (d) STE emission mechanism. Reproduced from ref 44. Copyright 2023, Wiley.

### 3.2 Self-Trapped Exciton Emission

An STE is an electron–hole pair that is localized or trapped by lattice distortions, in contrast to a free exciton that can propagate through the crystal.^22,42^ In simple words, lattice distortions create a local potential well that traps the exciton. In general, STE emission occurs in materials with a soft ionic lattice and strong interaction between electrons and lattice vibrations (electron–phonon coupling).^42,45^ Based on the reported literature, STE emissions are common in 0D and 1D metal halides, such as Cs_3_Cu_2_X_5_ and CsCu_2_X_3_.^5,46^ In this review article, most of the dopant ions to be discussed feature self-trapped excitonic emission. Hence, it is necessary to build a comprehensive understanding of the STE emission mechanism. The following subsections describe the fundamental features of an STE and outline the key characteristics for identifying the presence of STE emission in a material. In general, there are two types of STE: an intrinsic STE, which is inherent to the material, and an extrinsic STE, which is induced by external sources such as dopant ions.^47^ A schematic diagram of STE lattice distortion and its emission mechanism is illustrated in Figure 5c,d.

#### 3.2.1 Electron–Phonon Coupling

Electron–phonon coupling is the interaction between electrons and lattice vibration (phonons).^5^ In a solid material, atoms vibrate around their equilibrium positions. These vibrations can be described as continuous waves propagating throughout the lattice. The electron–phonon coupling is quantified by the Huang–Rhys factor (S), which is a dimensionless constant that describes the coupling strength of electrons and phonons in the lattice.^5,7^ In general, a high S factor indicates strong electron–phonon coupling strength and high possibility of STE formation.^22,34,48,49^ Upon absorption of a photon with energy equal to or greater than the bandgap, an electron is excited from the valence band to the conduction band, leaving behind a positively charged hole.^50,51^ The electrostatic attraction between the electron and hole leads to the formation of a bound electron–hole pair, known as an exciton.^50,51^ The exciton perturbs the local electronic charge distribution, altering the electrostatic interactions with the surrounding ions. As a result, the neighboring ions shift slightly from their equilibrium positions, producing a local lattice distortion through electron–phonon coupling.^34,49^ When electron–phonon coupling is sufficiently strong, the exciton induces significant lattice relaxation, resulting in a local lattice distortion. The lattice distortion creates a local potential well that traps the exciton within its own self-induced distortion, forming an STE.^51^ Upon radiative recombination, the STE emits light through multiple vibronic transitions, giving rise to a broadband emission, large Stokes shifts, and relatively long radiative decay lifetime.^34,49^

#### 3.2.2 Broadband PL Emission

To understand the origin of the broadband emission in STE materials, it is necessary to consider the relationship between two concepts, namely electronic transitions and electron–phonon coupling.^5,50^ First, it must be emphasized that electrons are restricted to discrete energy levels, meaning they can only occupy certain allowed energy levels.^5,50^ These energy levels arise from the quantum mechanical nature of electrons. The transitions between these energy levels correspond to photon absorption (excitation) or emission of photons with energies equal to the difference between the energy levels. Second, one must realize that atoms in a solid are in a constant state of vibration that are quantized as phonons.^5,50^ The interaction between the electrons and phonons also known as electron–phonon coupling is actually the main cause behind the broadband emission.^5,50^ In essence, strong electron–phonon coupling distributes the energy of an electronic transition across multiple vibrational states, resulting in the emission of photons with a range of energies which cause the broadband emission.^5,50^ In contrast, weak electron–phonon coupling results in a narrow emission because most of the transition energy is emitted without much interaction with phonons. As a result, the emitted photons have almost identical energies, resulting in a narrow emission or in some cases, a sharp, well-defined spectral line, as observed in trivalent Ln^3+^ ions.^5,50^

#### 3.2.3 Large Stokes Shift

Stokes shift is the energy difference between the absorption and emission maxima (Figure 6a,b). A question arises as to why 0D metal halides exhibit a large Stokes shift.^37^ This behavior is attributed to the lattice distortion and self-trapping of free excitons, which lowers their energy.^49,52^ In fact, the PL emission observed is from the self-trapped excitons, which lost some of their energy during the self-trapping process. Therefore, the Stokes shift corresponds to the energy difference between the absorption energy of the free excitons and the subsequent PL emission from the self-trapped excitons. From these observations, it is apparent that the large Stokes shift is one of the fundamental differences between 0D Zn metal halides and CsPbBr_3_ perovskites.

**Figure 6:**
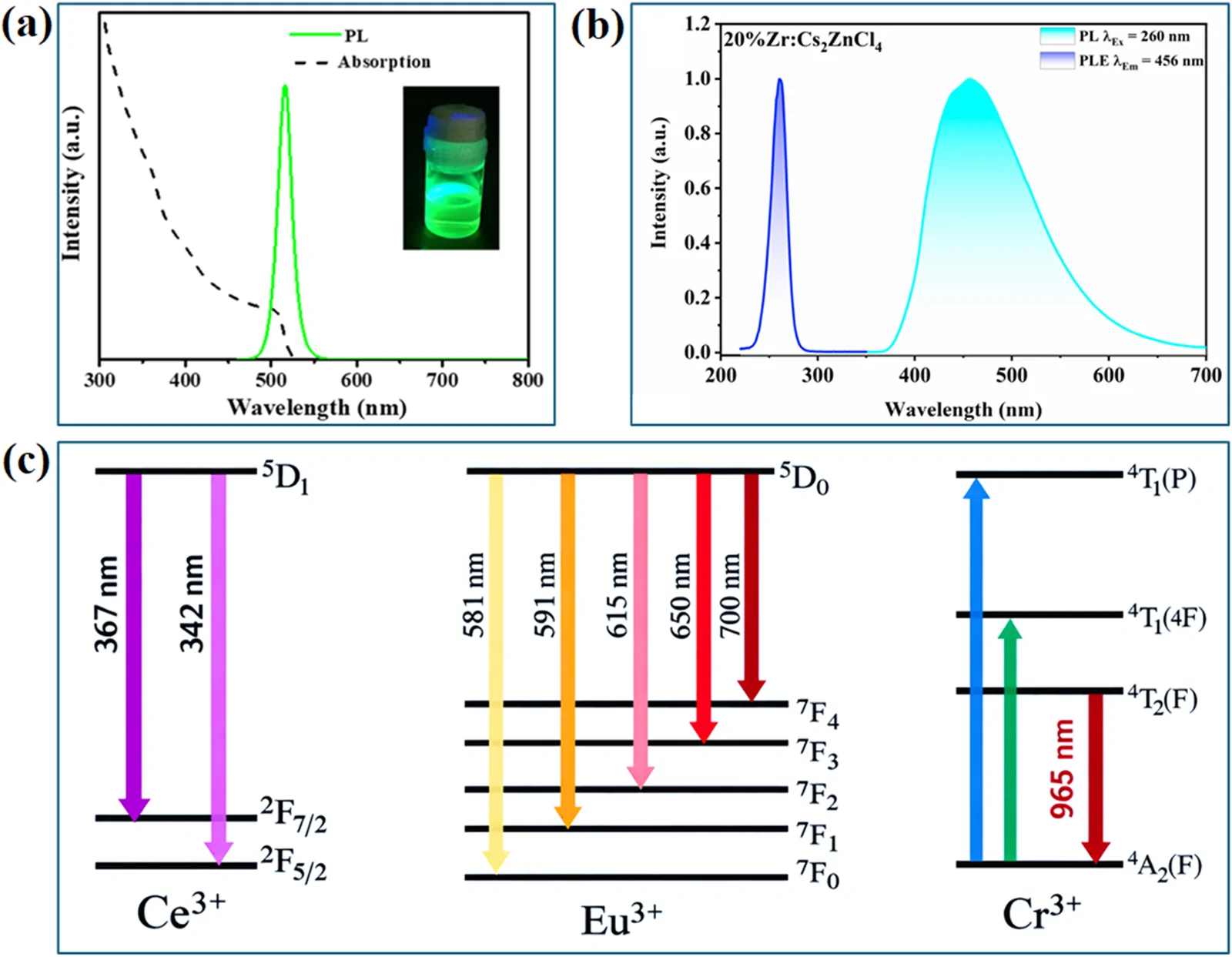
(a) Narrow Stokes Shift in CsPbBr_3_ perovskites. Reproduced from ref 53. Available under a CC-BY 4.0 license. Copyright 2019, Optica Publishing Group. (b) Large Stokes shift in Zr^4+^-doped Cs_2_ZnCl_4._ Reproduced from ref 22. Available under a CC-BY 4.0 license. Copyright 2024, MDPI. (c) Ion-luminescent emission and electronic transitions in Ce^3+^, Eu^3+^ and Cr^3+^ ions. Reproduced from ref 5. Available under a CC-BY 4.0 license. Copyright 2025, Wiley.

#### 3.2.4 Long Radiative Decay Lifetime

The radiative decay lifetime of self-trapped excitons is the average time required for self-trapped excitons to recombine radiatively and emit photons. The long radiative decay lifetime of self-trapped excitons arises from their localization in a deep potential well formed by lattice distortion and from intersystem crossing, which is associated with spin-forbidden transitions.^19,42^ It is worth clarifying that spin-forbidden transitions should not be understood in an absolute sense but rather as transitions with a low probability of occurring due to violation of the spin selection rule.^5,34^ As a result, fewer photons are emitted per unit time, leading to slow emission rate and longer radiative decay lifetime. This behavior reflects the inverse relationship between the transition probability and the lifetime (τ ∝ 1/*A*).

#### 3.2.5 Exciton Binding Energy

Exciton binding energy is the amount of energy needed to pull apart an electron and a hole that are held together by Coulombic attraction.^18,22,50^ In a free exciton, the binding between the electron and hole arises primarily from their Coulombic attraction. In contrast, a self-trapped exciton experiences strong interactions with lattice vibrations (phonons), causing the surrounding atoms to shift and create a self-induced potential well.^18,22,50^ Lattice distortion caused by self-trapping lowers the exciton’s total energy and confines the electron and hole to a small region of the crystal. This strong localization increases the overlap between the electron and hole, enhancing their Coulombic attraction and making the exciton more tightly bound.^18,22,50^

#### 3.2.6 Exciton Recombination Process

An exciton forms when an electron is excited to a higher-energy state in the conduction band, leaving behind a hole in a lower-energy state in the valence band.^18,50,51^ The electron and hole are attracted to each other by Coulombic attraction, which is electrostatic in nature because the electron is negatively charged and the hole is positive.^18,50,51^ Recombination occurs when the electron moves into a hole and fills it, causing the exciton to disappear. The energy stored in the exciton is released either as a photon through a radiative process or phonons through a nonradiative process.^18,50,51^

### 3.3 Ion-Luminescence Emission

Ion-luminescence is a PL emission mechanism in which ions emit photons via radiative relaxation upon excitation.^5,34^ In this process, excitation promotes the electrons from the ground state to higher excited states.^5,34^ Then, the electrons undergo nonradiative relaxation to the lowest excited level. After that, electrons undergo radiative relaxation to the ground state, resulting in the emission of photons. In general, ion-luminescence is common in transition metals (Mn^2+^) with partially filled d orbitals or trivalent Ln^3+^ ions with partially filled f orbitals.^5,34^ Figure 6c lists some ions that exhibit this emission mechanism, along with their corresponding energy levels and electronic transitions. From a solid-state physics perspective, the splitting of energy levels arises from electron–electron interactions and spin–orbit coupling, which cause the degenerate electronic states to separate into multiple discrete and quantized energy levels.^5,34^

## 4 Electronic Properties and Electronic Transitions

This section discusses the density of states and the contributing orbitals with regard to the electronic transitions of Zn metal halides. It is important to keep in mind that the computed bandgap value of an exact same material may differ from one study to another due to inherent errors in DFT calculations. Nonetheless, theoretical calculations provide insights into how doping affects the electronic structure of Zn metal halides, thereby modulating their luminescent properties.

### 4.1 Density of States

By analyzing the density of states, crystal structure and the electronic configuration of Cs_2_ZnBr_4_, one can extract that the electronic and optical properties largely depend on the isolated [ZnX_4_]^2–^ polyhedra, which are made of Zn^2+^ cations and X^–^ halide ions (Figure 7a–d).^16,40^ The Cs^+^ ions function as spatial isolators to separate the [ZnX_4_]^2–^ polyhedra.^21^ The Cs^+^ orbitals do not contribute to the density of states.^54^ The main orbitals that contribute to the valence band maximum (VBM) are Zn-3d and Cl-3p states, whereas the conduction band minimum (CBM) is composed of Zn-4s and Cl-3p states.^18,55^

**Figure 7:**
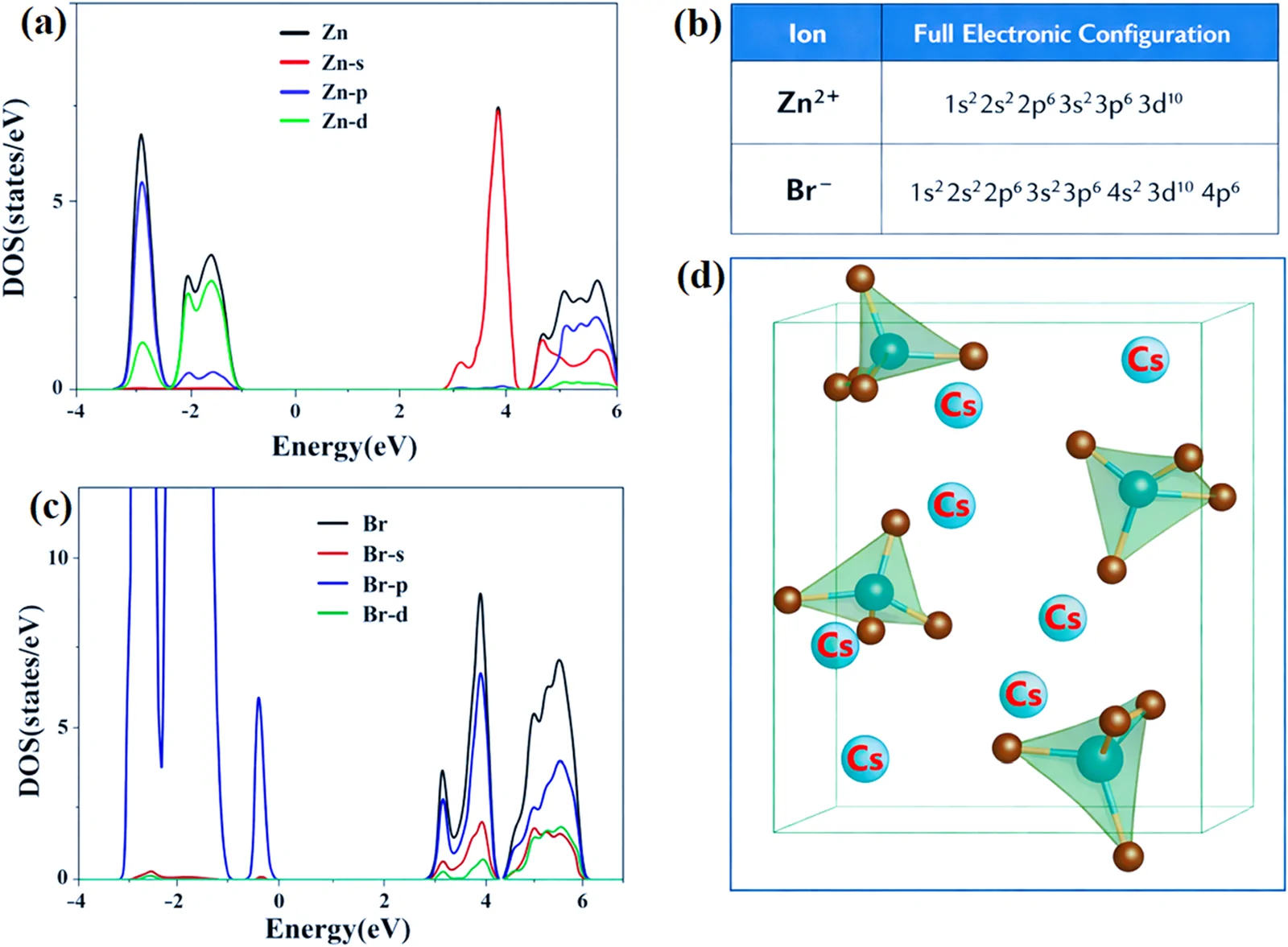
(a) Partial density of states of Zn. (b) Electronic configuration of Zn^2+^ and Br^–^. (c) Partial density of states of Br. Reproduced from ref 40. Copyright 2023, Wiley. (d) Crystal structure of Cs_2_ZnBr_4_. Reproduced from ref 16. Copyright 2020, Wiley.

### 4.2 d ↔ s Transitions

To answer the question of why Zn metal halides are classified as weakly emissive, the nature of the 3d → 4s transitions in Zn metal halides should be carefully analyzed.^48,56^ First, Zn^2+^ ions have a closed-shell 3d^10^ electronic configuration, which means d-d transitions are not possible in Zn^2+^ ions. For PL emission to occur, an electron must transition from the 3d^10^ orbitals to the vacant 4s orbital, forming a temporary 3d^9^4s^1^ state, and then relax radiatively from the 4s orbital back to the 3d^9^ orbitals to restore the 3d^10^ state and emit photons (Figure 8a).^15,57^ The weak PL emission in Zn metal halides is due to the vacant 4s orbital in Zn^2+^ ions being located at high energy levels. The divalent charge of Zn^2+^ ions exerts a strong effective nuclear attraction that pulls the 3d orbitals closer to the nucleus. As a result, the 3d orbitals are highly stabilized, meaning they are located at low energy levels. Because of this stabilization, the energy gap between the 3d and 4s orbital is too wide, preventing 3d → 4s transitions. In contrast, in Cs_3_Cu_2_X_5_, the weaker effective nuclear attraction implies that the 3d orbitals are not held tightly or highly stabilized.^15,57^ Therefore, it can be inferred that the 3d orbitals in Cu^+^ ions are close in energy to the vacant 4s orbital. This small energy gap makes the vacant 4s orbital accessible and allows electron transitions between the 3d and 4s orbitals (Figure 8b,c).^58^ Overall, the electronic transitions in Zn metal halides are metal-centered, involving 3d → 4s transitions, similar to those observed in Cs_3_Cu_2_X_5_ metal halides.^58^

**Figure 8:**
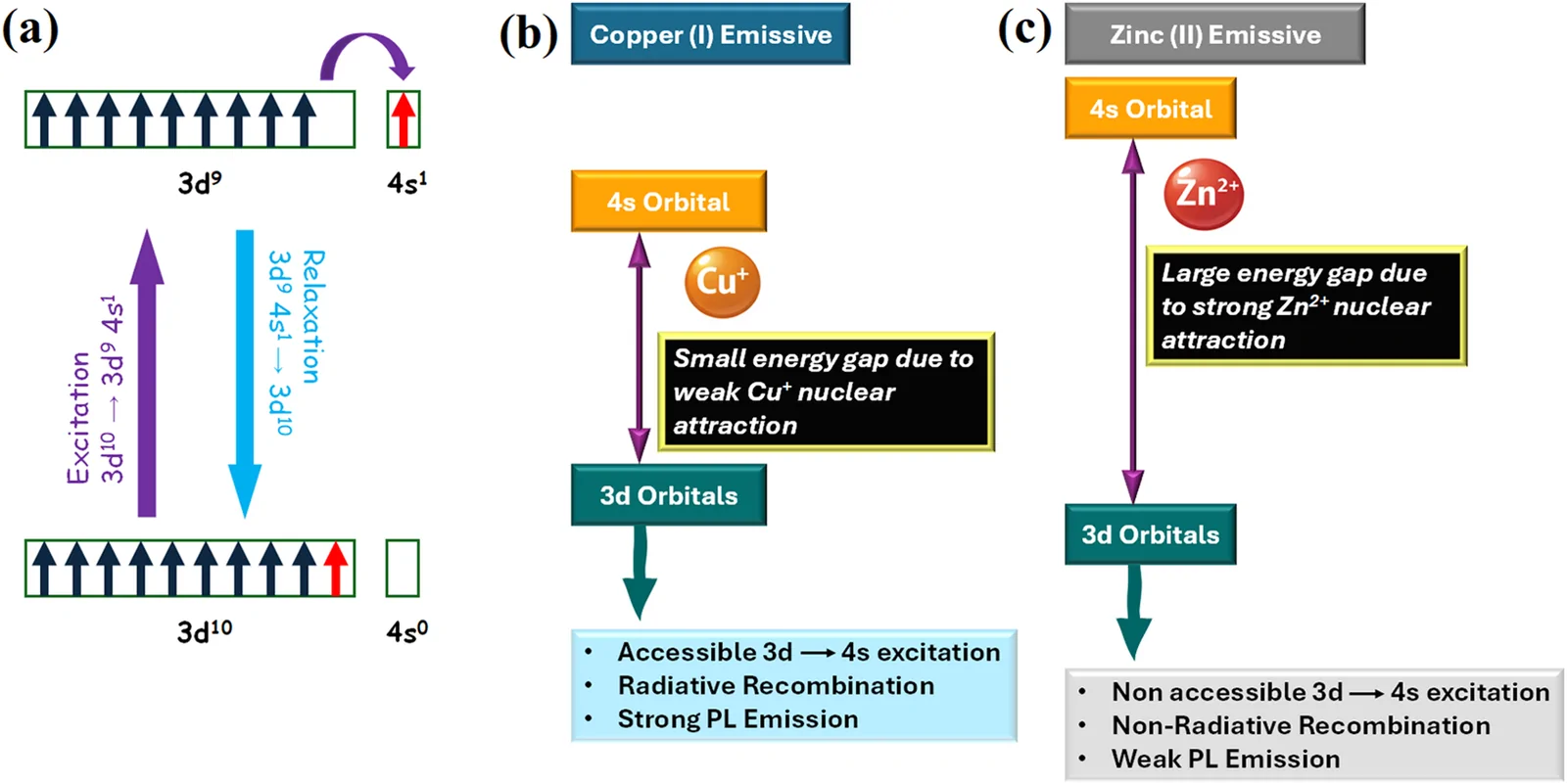
(a) 3d → 4s transitions. Reproduced from ref 46. Available under a CC-BY 4.0 license. Copyright 2026, Elsevier. (b) Relationship between effective nuclear attraction and energy gap between 3d and 4s in Cu^+^ ions. (c) Relationship between effective nuclear attraction and energy gap between 3d and 4s in Zn^2+^ ions.

### 4.3 s ↔ p Transitions

Ions with ns^2^ electronic configuration, such as Sb^3+^, Te^4+^, and Bi^3+^, are widely used as dopants to induce STE emission in 0D metal halides.^59^ In these ns^2^ ions, interactions between electrons and spin–orbit coupling cause the ns np excited configuration in ns^2^ ions to split into four energy levels: ^3^P_0_, ^3^P_1_, ^3^P_2_, and ^1^P_1_.^40^ The density of states of Sn^2+^ ions in Figure 9a reveals that the valence band is composed of 5s orbital, while the conduction band is constituted of empty 5p orbitals.^40^ Upon excitation, an electron is promoted from the 5s valence band to the 5p conduction band to create an ns^1^ np^1^ configuration in the excited states.^56^ In brief, the energy splitting of ns^2^ ions is presented in Figure 9b.^5,60^ The ^1^S_0_ → ^1^P_1_ transition is allowed according to the electric dipole and spin selection rules. The ^1^S_0_ → ^3^P_1_ transition is spin-forbidden because it involves a change in total spin.^40^ However, spin–orbit coupling mixes singlet and triplet states, giving the ^3^P_1_ state a small component of ^1^P_1_ character, making the transition partially allowed. On the other hand, the ^1^S_0_ → ^3^P_0_ and ^1^S_0_ → ^3^P_2_ transitions are both spin and electric-dipole forbidden.^40^

**Figure 9:**
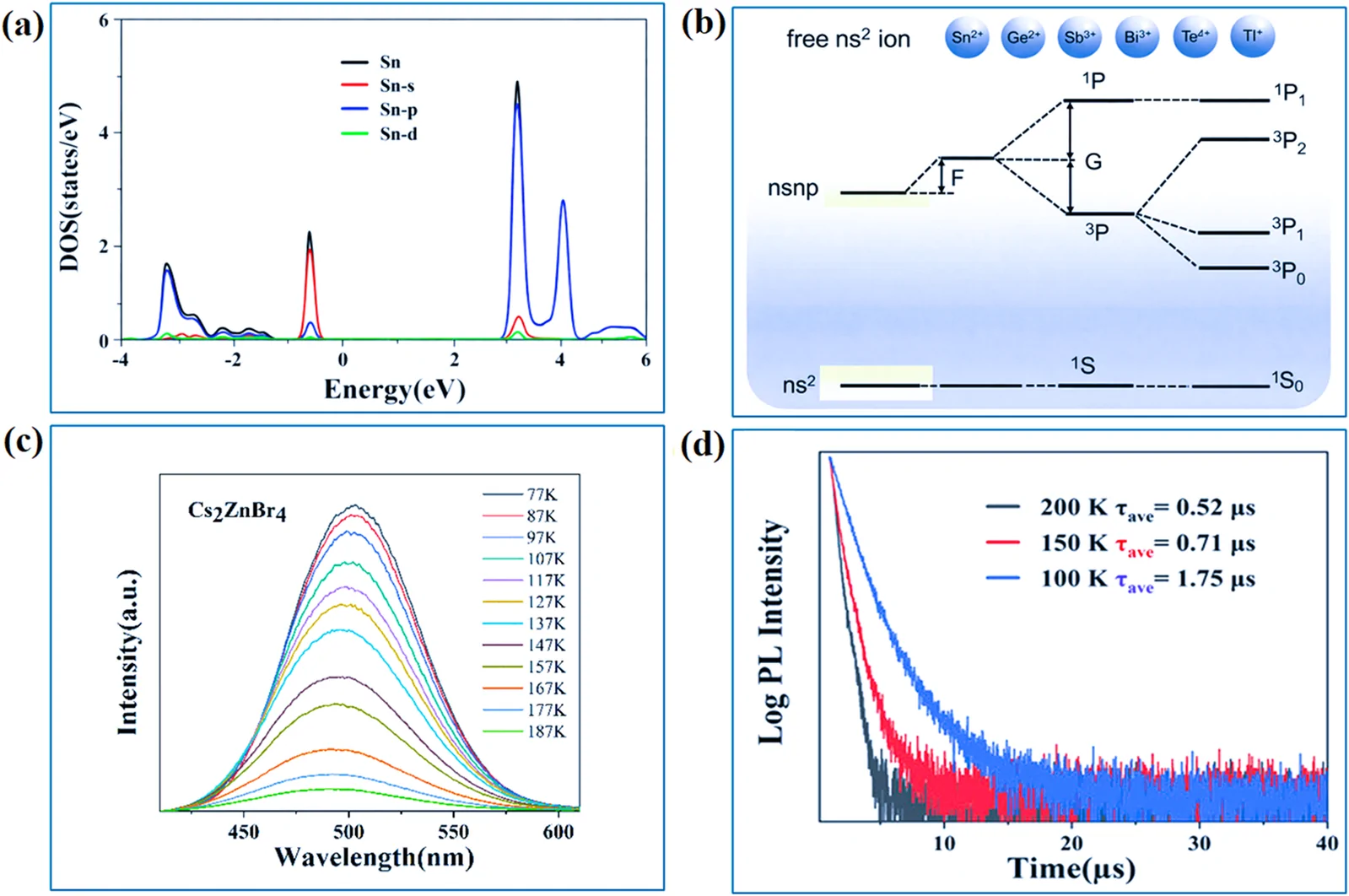
(a) Density of states image showing 5s → 5p electronic transitions in Sn^2+^ ions. (b) Energy splitting of ns^2^ ions to split into four energy levels: ^3^P_0_, ^3^P_1_, ^3^P_2_, and ^1^P_1_. Reproduced from ref 5. Available under a CC-BY 4.0 license. Copyright 2025, Wiley. (c) Temperature dependent PL measurements. (d) Time-resolved PL measurements. Reproduced from ref 40. Copyright 2023, Wiley.

### 4.4 Temperature Dependent PL Measurements

Following the discussion of the density of states and electronic transitions, the focus now shifts to temperature-dependent photoluminescence spectroscopy. This technique provides insights regarding the relationship between temperature and exciton recombination dynamics. At high temperatures, nonradiative recombination processes dominate, whereas at low temperatures these processes are suppressed.^40^ This explanation agrees with the results in Figure 9c, which show that the nonradiative recombination processes, which dominate at high temperatures (187 K), are greatly suppressed at lower temperatures (77 K) due to the inhibition of lattice vibrations.^40^ Furthermore, suppressed lattice vibrations due to reduced kinetic energy are confirmed by time-resolved photoluminescence (TRPL) measurements (Figure 9d).^40^ As the temperature decreases from 200 to 100 K, the average PL radiative decay lifetime increases from 0.52 to 1.75 μs, indicating an enhancement of radiative recombination rates.^40^

## 5 Luminescent Modulation via Dopant Ions

0D Zn metal halides with a wide bandgap are emerging as promising host materials to accommodate dopant ions which induce PL emission. In general, the PL emission in doped Zn metal halides can be attributed to two mechanisms: ion-luminescence in transition metal dopants such as Mn^2+^ or Cr^3+^ ions, or self-trapped excitons, which are observed in dopants such as Cu^+^, Zr^4+^, Bi^3+^, Sn^2+^, and Sb^3+^ ions. From a band-structure perspective, dopants are often grouped into two broad categories based on how they alter the host electronic states. Dopants such as Mn^2+^ ions introduce intragap states or midgap states that are electronic levels located within the bandgap between the valence and conduction bands. In simple words, dopants like Mn^2+^ ions create localized states without causing a significant change in the electronic band structure. In contrast, dopants like Ce^3+^ ions that interact strongly with the host electronic states and hybridize with the valence or conduction bands, leading to modifications in the valence or conduction band positions. Overall, dopant ions introduce additional absorption and energy levels, tune the bandgap, and enhance radiative recombination and PLQY.

### 5.1 Violet Emission

Luminescent materials that emit ultraviolet light are used in various applications, such as sterilization, dental sealant curing, dermatological treatments, and anticounterfeiting labels.^1,5^ At present, commercial UV-emitting devices are based on III–V semiconductors, which require sophisticated fabrication involving a silicon substrate, strong-vacuum conditions, and high temperatures.^1,5^ In contrast, Zn metal halides can be synthesized using low-cost and low-temperature solution-processable methods.^1,20^ As discussed earlier, Zn metal halides can accommodate a wide variety of dopant ions in their lattice. Upon successful doping, new luminescent properties can be induced into Zn metal halides.^1,20^ For instance, Ce^3+^ ions are widely used as dopants to induce UV emission into host materials. As shown in Figure 10a, Ce^3+^-doped Cs_2_ZnBr_4_ exhibits two asymmetric peaks centered at 342 nm (5d^1^ → ^2^F_5/2_) and 367 nm (5d^1^ → ^2^F_7/2_).^1,61^

**Figure 10:**
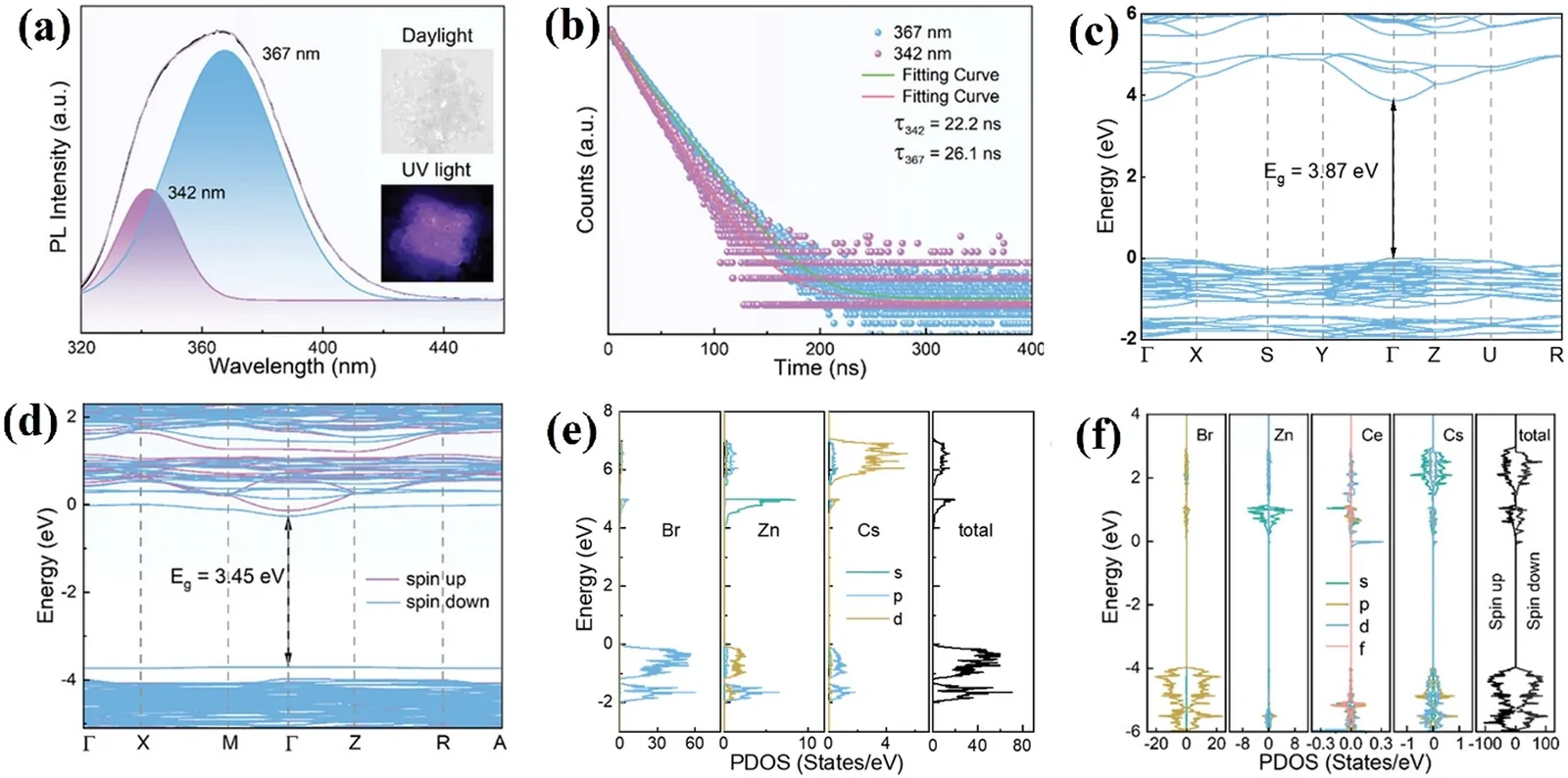
(a) PL spectra of Ce^3+^-doped Cs_2_ZnBr_4_. (b) TRPL of Ce^3+^-doped Cs_2_ZnBr_4_. (c) Band structure of undoped Cs_2_ZnBr_4_. (d) Band structure of Ce^3+^-doped Cs_2_ZnBr_4_. (e) PDOS of Cs_2_ZnBr_4_. (f) PDOS Ce^3+^-doped Cs_2_ZnBr_4_. Reproduced from ref 1. Copyright 2025, Wiley.

It is important to point out that Ce^3+^-based materials are known to exhibit high PLQY. In fact, Ce^3+^-doped Cs_2_ZnBr_4_ exhibits high PLQY around 97%.^1,61^ The high PLQY in Ce^3+^-based materials is due to their spin- and parity-allowed 5d-4f transitions.^1,61^ Moreover, Ce^3+^-doped Cs_2_ZnBr_4_ reveals a radiative decay lifetime around 22–26 ns, which is close to the reported values in trivalent lanthanide metal halides such as Cs_3_CeCl_6_ or Cs_3_CeBr_6_ (Figure 10b). The reason behind the similar radiative decay lifetime in Ce^3+^-doped Cs_2_ZnBr_4_ and Cs_3_CeX_6_ (X = Cl, Br, or I) is that in these materials, the electronic transitions originate from spin- and parity-allowed 5d-4f transitions.^1,61^ A short radiative decay lifetime means that more photons are emitted per unit time because the higher radiative decay rate (1/τ) causes the excited ions to return to the ground state faster. In addition, a short radiative decay lifetime can also indicate an allowed electronic transition with high oscillator strength. For theoretical context, oscillator strength is a measure of the probability of an electronic transition between two energy levels. A high oscillator strength means that the transition is more probable and results in a strong absorption or emission intensity accompanied by a short radiative decay lifetime. For the sake of brevity, the band structure and PDOS results are presented in Figure 10c–f, and the results are concisely interpreted Table 2.^1^

**Table 2:** Band Structure and PDOS of Cs_2_ZnBr_4_ and Ce^3+^-Doped Cs_2_ZnBr_4_

material	band gap (eV)	band gap type	(VBM)	(CBM)	refs
Cs_2_ZnBr_4_	3.87	Direct (Γ point)	Br-4p, Zn-3d	Br-4p, Zn-4s	^1^
Ce^3+^-doped Cs_2_ZnBr_4_	3.45	Direct (Γ point)	Br-4p, Ce-4f	Br-4p, Ce-5d	^1^

### 5.2 Blue Emission

Blue emission can be induced in Zn metal halides using dopant ions such as Cu^+^, Bi^3+^, and Zr^4+^.^20,22,62^ According to recent studies, Cs_3_Cu_2_I_5_ exhibits deep blue emission centered at 443 nm and a high PLQY of ∼72%.^63,64^ Nonetheless, Cu^+^ ions tend to oxidize to Cu^2+^ when stored under ambient conditions over a long period of time.^63^ To avoid the oxidation of Cu^+^ to Cu^2+^ ions, alternative stable dopant ions such as Bi^3+^ or Zr^4+^ are recommended.^20,62^ These stable dopants can generate a broadband blue emission via STE emission arising from [BiX_4_]^−^ or [ZrX_4_]^2–^ polyhedra.^20,62^ This subsection analyzes and compares the impact of Cu^+^, Bi^3+^, and Zr^4+^ dopants on the optical and electronic properties of Zn metal halides.

#### 5.2.1 Cu^+^-Doped Zn Metal Halides

Despite the differences in oxidation states, Cu^+^ and Zn^2+^ ions show similar chemical characteristics because both ions belong to the same row of the transition metals and share comparable ionic radii and coordination behavior. As shown in Figure 11a,b, Cs_2_ZnBr_4_ as an impractical blue emission with a PLQY of 3.6% and even appears nonemissive under direct observation with the human eye.^20^ Upon doping with Cu^+^ ions, Cs_2_ZnBr_4_ reveals an intense absorption peak at 311 nm and a strong blue emission centered at 450 nm with a high PLQY of 65.3%.^20^ These results confirm that the strong blue emission originates from the substitution of tetrahedrally coordinated Zn^2+^ ions by Cu^+^ ions, resulting in the formation of luminescent [CuBr_4_]^3–^ polyhedra. In addition, Cu^+^-doped Cs_2_ZnBr_4_ shares similarities with Cs_3_Cu_2_Br_5_ because the optical properties of both materials are largely governed by the [CuBr_4_]^3–^ luminescent polyhedra.^20,37^ As a result, both Cu^+^-doped Cs_2_ZnBr_4_ and Cs_3_Cu_2_Br_5_ exhibit triplet STE emission, which is characterized by three main features, namely a large Stokes shift, broadband emission, and a long microsecond radiative decay lifetime, as seen in other 0D metal halides such as Cs_3_Cu_2_X_5_ (Figure 11c).^19,20^

**Figure 11:**
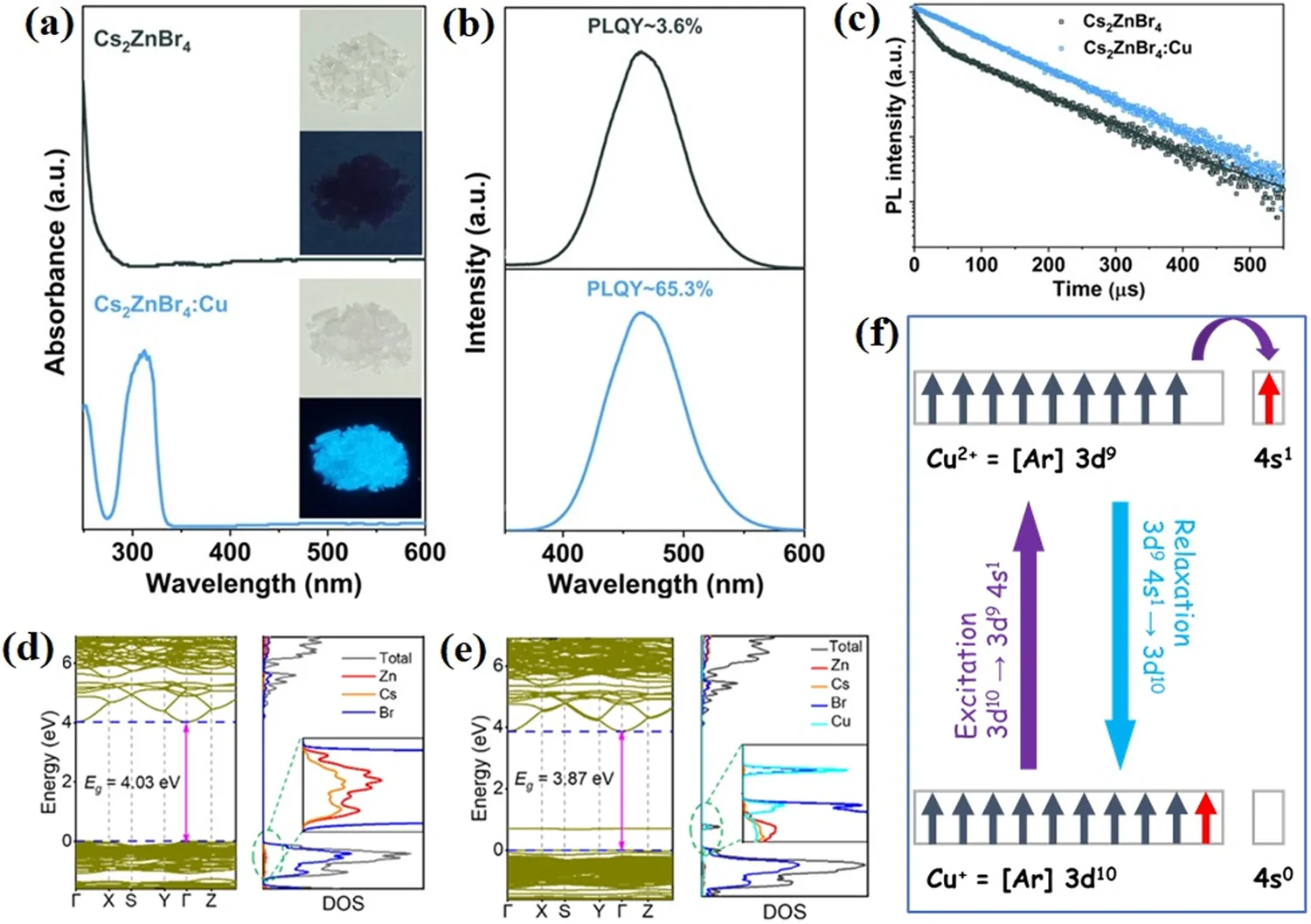
(a) Absorbance spectra of Cs_2_ZnBr_4_ and Cu^+^-doped Cs_2_ZnBr_4_. (b) PL emission of Cs_2_ZnBr_4_ and Cu^+^-doped Cs_2_ZnBr_4_. (c) TRPL of Cs_2_ZnBr_4_ and Cu^+^-doped Cs_2_ZnBr_4_. Reproduced from ref 16. Copyright 2020, Wiley. (d) Band structure and DOS of Cs_2_ZnBr_4_. (e) Band structure and DOS of Cu^+^-doped Cs_2_ZnBr_4_. Reproduced from ref 3. Copyright 2024, American Chemical Society. (f) 4s ↔ 3d transitions in [CuBr_4_]^3–^ units. Reproduced from ref 46. Available under a CC-BY 4.0 license. Copyright 2026, Elsevier.

The band structure and DOS of Cs_2_ZnBr_4_ and Cu^+^-doped Cs_2_ZnBr_4_ are presented in Figure 11d,e and summarized in Table 3 to illustrate the impact of Cu^+^ dopants on the electronic and optical properties of Cs_2_ZnBr_4_.^20^ As expected, doping with Cu^+^ ions reduced the bandgap from 4.03 to 3.87 eV.^20^ In addition, Cu^+^ dopants introduced new emission levels through 4s → 3d transitions within the [CuBr_4_]^3–^ polyhedra (Figure 11f). Moreover, both Cs_2_ZnBr_4_ and Cu^+^-doped Cs_2_ZnBr_4_ show a flat valence band, a characteristic of 0D metal halide systems, indicating the presence of strongly localized holes.^3^ Based on these results, it is evident that Cu^+^-doped Cs_2_ZnBr_4_ shows strong blue emission, but the problem is that Cu^+^ ions are susceptible to oxidation to Cu^2+^ ions.^23,41^ Consequently, Cu^+^-doped materials are prone to undergoing phase transformation and losing their luminescent properties. In fact, when Cs_3_Cu_2_Br_5_ oxidizes, it undergoes a phase transformation to Cs_2_CuBr_4_, which has weak luminescence.^23,41^

**Table 3:** Band Structure and DOS of Cs_2_ZnBr_4_ and Cu^+^-Doped Cs_2_ZnBr_4_

material	band gap (eV)	band gap type	(VBM)	(CBM)	refs
Cs_2_ZnBr_4_	4.03	Direct (Γ point)	Br-4p, Zn-3d	Br-4p, Zn-4s	^16^
Cu^+^-doped Cs_2_ZnBr_4_	3.87	Direct (Γ point)	Br-4p, Cu-3d	Br-4p, Cu-4s	^16^

#### 5.2.2 Zr^4+^-Doped Zn Metal Halides

From the standpoint of stability, Zr^4+^ ions are considered more suitable dopants than Cu^+^ ions. The reason is that Zr^4+^ ions are stable in air, unlike Cu^+^ ions which tend to oxidize to Cu^2+^ ions when exposed to air over a long period of time. As shown in Figure 12a,b, incorporating Zr^4+^ ions in Cs_2_ZnCl_4_ distorts the lattice, elongates the metal-halide bond length and compresses the bond angles due to the larger Zr^4+^ ionic radius compared to Zn^2+^ ions.^22^ Simultaneously, the lattice distortions led to stronger electron–phonon coupling, which is essential for the formation of an STE. Moreover, introduction of Zr^4+^ dopants decreased the bandgap of Cs_2_ZnCl_4_ from 4.8 to 4.15 eV (Figure 12c,d).^22^ To elucidate the electronic transitions in Zr^4+^-doped Cs_2_ZnCl_4_, one must keep in mind that neutral Zr has an electronic configuration of ([Kr] 4d^2^ 5s^2^), when neutral Zr loses four electrons, it forms Zr^4+^ ([Kr] 4d^0^) with vacant 4d orbitals.^47,65^ The vacant Zr-4d orbitals contribute negligibly to the VBM at the Fermi level but dominate the CBM. The other piece of the puzzle can be obtained by examining the DOS of Cl, in which the 3p orbitals of Cl constitute the VBM (Table 4).^47,65^ Therefore, when these two pieces of information are combined, it can be inferred that the electronic transitions in the [ZrCl_4_] units are dominated by ligand-to-metal charge transfer (Cl 3p → Zr 4d), as presented in Figure 12e,f.^22^ These results elucidate the origin of the high excitation energy (254 nm) observed in both Cs_2_ZrCl_6_ and Zr^4+^-doped Cs_2_ZnCl_4_. The energy gap between the Cl 3p orbitals and empty Zr 4d orbitals is very large. As a result, the charge transfer process requires high-energy UV photons to overcome the wide energy gap and thus explains why the excitation peak is in the UVC region around 254 nm.

**Figure 12:**
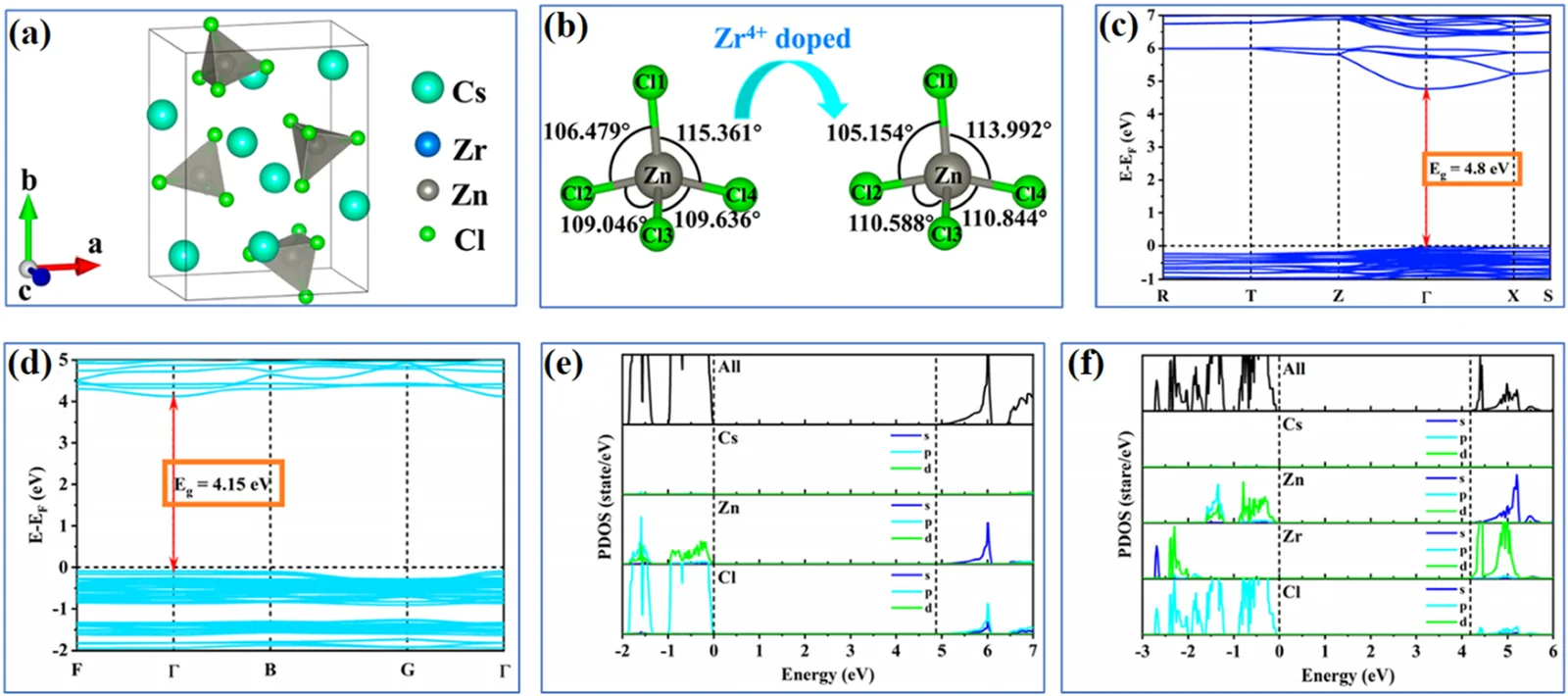
(a) Zr^4+^-doped Cs_2_ZnCl_4_ crystal structure. (b) Bond angles before and after doping. (c) Undoped Cs_2_ZnCl_4_ band structure. (d) Zr^4+^-doped Cs_2_ZnCl_4_ band structure. (e) Undoped Cs_2_ZnCl_4_ PDOS. (f) Zr^4+^-doped Cs_2_ZnCl_4_ PDOS. Reproduced from ref 22. Available under a CC-BY 4.0 license. Copyright 2024, MDPI.

**Table 4:** Band Structure and DOS of Cs_2_ZnCl_4_ and Zr^4+^-Doped Cs_2_ZnCl_4_

material	band gap (eV)	band gap type	(VBM)	(CBM)	refs
Cs_2_ZnCl_4_	4.80	Direct (Γ point)	Zn-3d, Cl-3p	Zn-4s, Cl-3p	^22^
Zr^4+^-doped Cs_2_ZnCl_4_	4.15	Direct (Γ point)	Cl-3p	Zr-4d	^22^

Upon excitation at 254 nm, Zr^4+^-doped Cs_2_ZnCl_4_ shows a broadband blue emission centered at 456 nm with a large Stokes shift of 197 nm and a high PLQY of 89.67% (Figure 13a).^22^ Temperature-dependent PL measurements reveal that the blue emission in Zr^4+^-doped Cs_2_ZnCl_4_ is highly dependent on the intensity of the electron–phonon interaction. In Figure 13b, at low temperatures between 50K and 150 K, the Zr^4+^-related STE emission is suppressed because the lattice distortion is too weak to generate an STE emission.^22^ However, as the temperature increases above 150 K, the lattice vibrations and electron–phonon interactions start to get strong enough to generate the Zr^4+^-related STE emission. Overall, Zr^4+^-doped Cs_2_ZnCl_4_ exhibits broadband blue emission, large Stokes shift, high Huang–Rhys factor (*S*) of 40.34, strong electron–phonon coupling of 40.89 meV, and a high exciton binding energy (*E*_b_) of 481.98 meV, all of which are strong indications of STE emission (Figure 13c,d).^22^ Most importantly, it is also noted that Zr^4+^-doped Cs_2_ZnCl_4_ shares similar optical properties with Cs_2_ZrCl_6_.^47,65^

**Figure 13:**
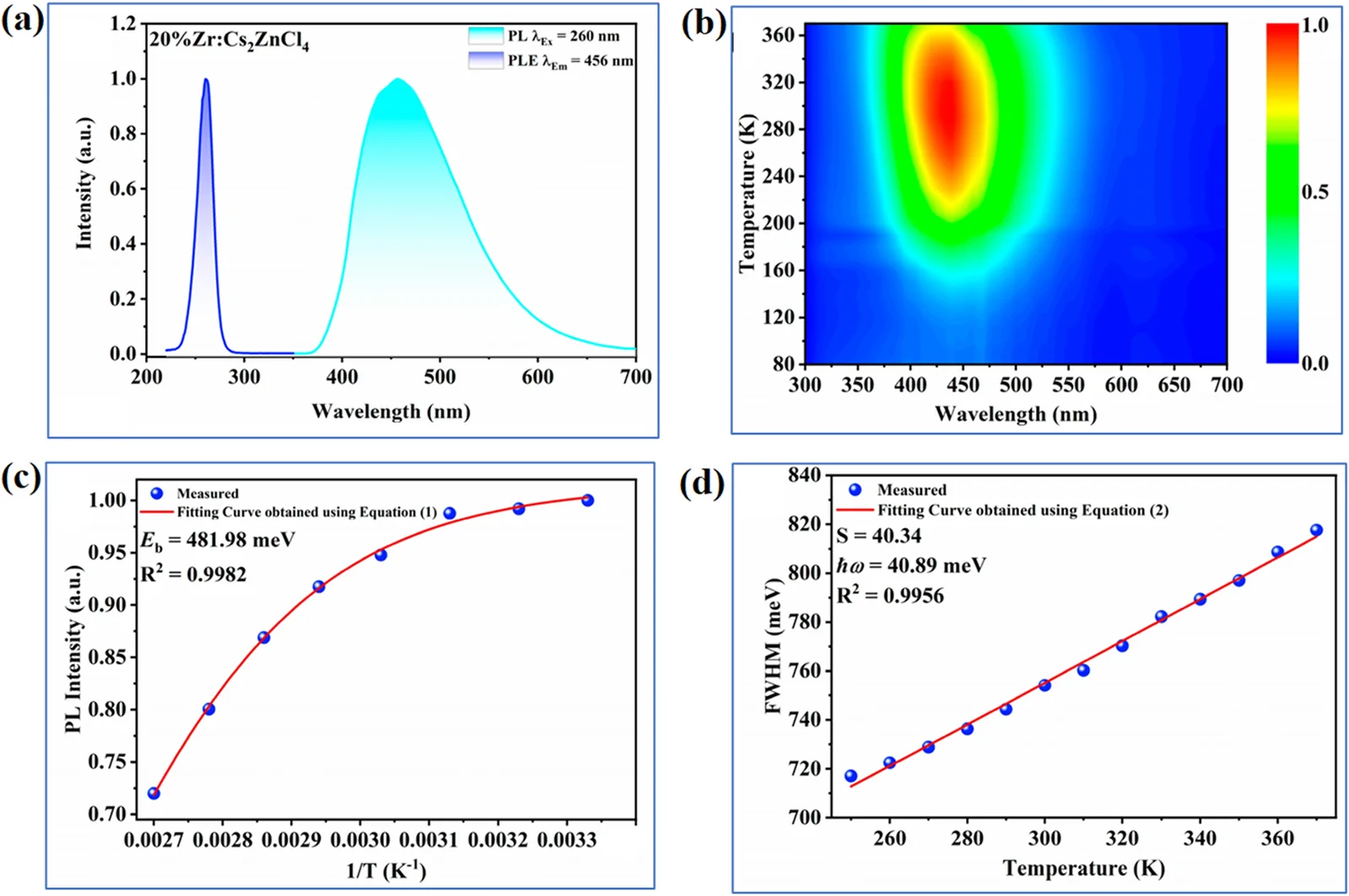
(a) PL and PLE of Zr^4+^-doped Cs_2_ZnCl_4_. (b) Temperature-dependent PL mapping of Zr^4+^-doped Cs_2_ZnCl_4_. (c) Exciton binding energy of Zr^4+^-doped Cs_2_ZnCl_4_. (d) Huang–Rhys factor (S) of Zr^4+^-doped Cs_2_ZnCl_4_. Reproduced from ref 22. Available under a CC-BY 4.0 license. Copyright 2024, MDPI.

#### 5.2.3 Bi^3+^-Doped Zn Metal Halides

Bi^3+^ ions are widely used to induce blue emission in A_2_BX_6_ double perovskites such as Cs_2_ZrCl_6_ or Cs_2_SnCl_6_.^66^ In Cs_2_ZnCl_4_, Bi^3+^ ions introduced an impurity state that reduced the bandgap and localized the band edges.^62^ This occurs when some [ZnCl_4_]^2–^ tetrahedra are replaced by [BiCl_4_]^−^ tetrahedra to induce blue emission at 445 nm under 365 nm excitation with a PLQY around 57% (Figure 14a,b).^62^

**Figure 14:**
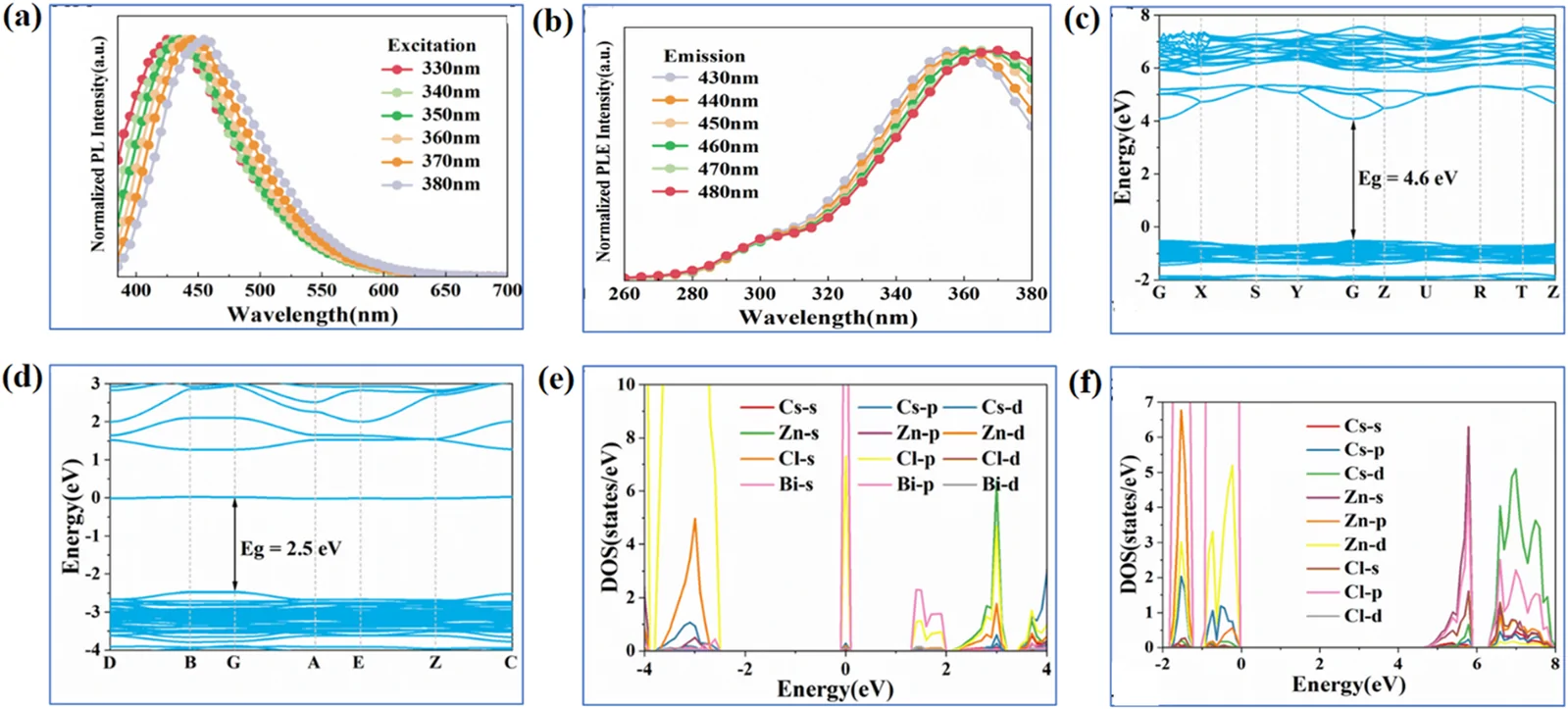
(a) PL spectra of Bi^3+^-doped Cs_2_ZnCl_4_. (b) PLE spectra of Bi^3+^-doped Cs_2_ZnCl_4_. (c) Band structure of undoped Cs_2_ZnCl_4_. (d) Band structure of Bi^3+^-doped Cs_2_ZnCl_4_. (e) DOS of Bi^3+^-doped Cs_2_ZnCl_4_. (f) DOS of undoped Cs_2_ZnCl_4_. Reproduced from ref 62. Copyright 2023, Royal Chemical Society.

In contrast to Cu^+^ and Zr^4+^, whose excitation peaks are in the high-energy UV region, the excitation peak of Bi^3+^ occurs in the UVA wavelength region around 365 nm. Thus, excitation at 365 nm allows Bi^3+^-doped Cs_2_ZnCl_4_ to emit blue light while requiring lower-energy UV photons compared to deep-UV excitation. In addition, materials that can be excited at 365 nm are more suitable and compatible with the existing LED hardware, such as optical lasers or UVA LED chips. Moving on to the electronic band structure (Figure 14c,d), pristine Cs_2_ZnCl_4_ has a direct bandgap of 4.6 eV with the VBM dominated by Cl-3p and Zn-3d orbitals and the CBM by Zn-4s and Cl-3p orbitals.^62^ As expected, Bi^3+^ doping causes a significant change in the band structure, by introducing a midgap 2.5 eV above the VBM. In addition, the VBM composition of Bi^3+^-doped Cs_2_ZnCl_4_ is largely dominated by Bi 6s and Cl-3p orbitals, while the CBM is composed of Bi-6p and Cl-3p orbitals (Figure 14e,f) and (Table 5).^62^

**Table 5:** Band Structure and DOS of Cs_2_ZnCl_4_ and Bi^3+^-Doped Cs_2_ZnCl_4_

material	band gap (eV)	band gap type	(VBM)	(CBM)	refs
Pristine Cs_2_ZnCl_4_	4.6	Direct (Γ point)	Cl-3p, Zn-3d	Zn-4s, Cl-3p	^62^
Bi^3+^-doped Cs_2_ZnCl_4_	2.5 (midgap state)	Direct (Γ point)	Bi-6s, Cl-3p	Bi-6p, Cl-3p	^62^

### 5.3 Green Emission

Divalent Mn^2+^ ions in tetrahedral coordination are known to emit green PL around 520–550 nm, depending on coordination geometry, crystal field strength, and ligand environment. The PL emission in Mn^2+^ ions is attributed to ion-luminescence through d-d transitions, in which the Mn ions emit photons via radiative relaxation upon irradiation (Figure 15a,b).^34^ Ion-luminescence entails three steps: excitation of electrons to higher energy levels, nonradiative relaxation to the lowest excited state, and radiative relaxation back to the ground state.^5^

**Figure 15:**
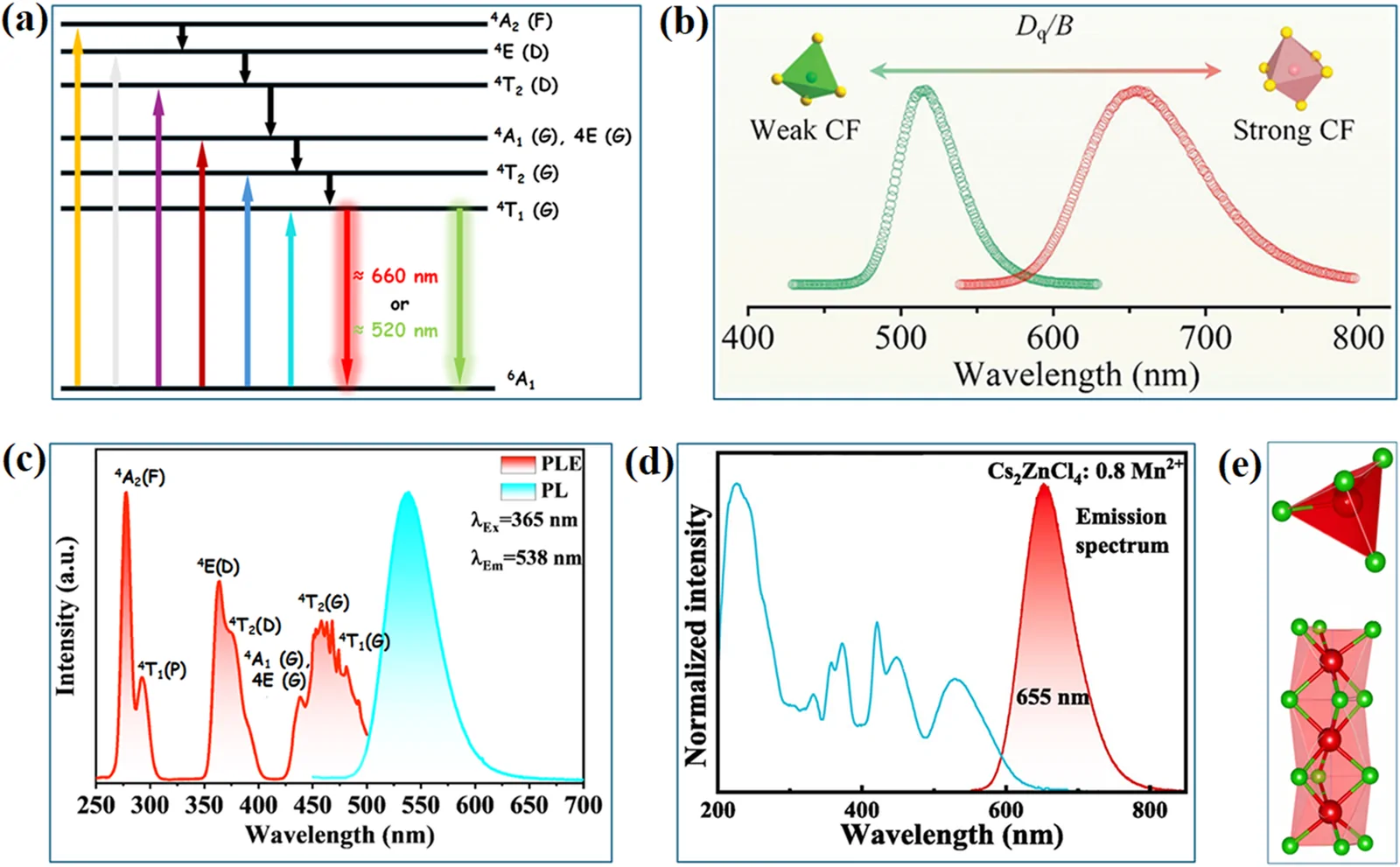
(a) d-d transitions in Mn^2+^ ions. (b) The influence of local coordination and crystal field on PL emission. Reproduced from ref 67. Copyright 2024, Wiley. (c) PL and PLE spectra of Mn^2+^-doped Zn halides. Reproduced from ref 68. Available under a CC-BY 4.0 license. Copyright 2024, MDPI. (d) PL and PLE spectra of Mn^2+^-doped Cs_2_ZnCl_4_. (e) [MnX_4_]^2–^ and [Mn_3_Cl_12_]^6–^ units. Reproduced from ref 69. Copyright 2023, Elsevier.

The optical properties in Mn^2+^-doped materials are largely dependent on the coordination around the Mn^2+^ ions. In general, tetrahedral coordinated Mn^2+^ ions show green emission, while octahedral coordinated show red emission. The PL and PLE in Figure 15c, show multiple excitation peaks at 298, 314, 382, 394, 486 nm, corresponding to the ^6^A_1_ → ^4^A_2_ (F), ^6^A_1_ → ^4^T_1_ (P), ^6^A_1_ → ^4^E (D), ^6^A_1_ → ^4^T_2_ (D), ^6^A_1_ → ^4^A_1_ (G), ^4^E (G), and ^6^A_1_ → ^4^T_1_ (G) d-d transitions in [MnX_4_]^2–^ tetrahedra.^68^ Upon excitation at any of these wavelengths, Mn^2+^-doped Zn metal halides show the same green PL emission with consistent shape and position irrespective of the excitation wavelength, which indicates that the PL emission is localized in [MnX_4_]^2–^ units.^34^ For theoretical context, the multiple excitation peaks in Mn^2+^-doped Zn metal halides arise from the electronic structure of Mn^2+^ ions and their d-d transitions.^34^ In isolated Mn^2+^ ions, the d orbitals are degenerate, meaning all d orbitals have the same energy. On the other hand, in Mn^2+^-doped Zn metal halides, the tetrahedral coordinated halide ions induce a crystal field that splits the d orbitals of Mn^2+^ into distinct energy levels, between which electrons undergo d-d transitions. Each transition corresponds to a specific excitation peak, giving rise to the multiple excitation peaks as observed in the PLE spectra. Excessive Mn^2+^ doping results in Mn–Mn coupling, causing a red PL emission to appear in the PL spectra (Figure 15d).^34,68^ High Mn^2+^ dopant concentration induce structural modifications, whereby tetrahedrally coordinated [MnX_4_]^2–^ units convert into octahedrally coordinated [MnX_6_]^4–^ units, and groups of three octahedra connect through face-sharing to form linear trimeric [Mn_3_Cl_12_]^6–^ units, which exhibit red emission, as seen in octahedral CsMnCl_3_ (Figure 15e).^69^ The emission wavelength of Mn^2+^ ions is highly dependent on the host lattice crystal field environment, and that increasing the Mn^2+^ doping concentration causes the coordination environment to transition from a weak-field tetrahedral [MnCl_4_]^2–^ site to a strong-field octahedral [MnCl_6_]^4–^ site. Overall, Table 6 summarizes the DOS results of Mn^2+^-doped Cs_2_ZnCl_4_.^69^

**Table 6:** Band Structure and DOS of Cs_2_ZnCl_4_ and Mn^2+^-Doped Cs_2_ZnCl_4_

material	band gap (eV)	band gap type	(VBM)	(CBM)	refs
Pristine Cs_2_ZnCl_4_	4.6	Direct (Γ point)	Cl-3p, Zn-3d	Zn-4s, Cl-3p	^69^
Mn^2+^-doped Cs_2_ZnCl_4_	2.5 (midgap state)	Direct (Γ point)	Mn-3d, Cl-3p	Mn-3d, Cl-3p	^69^

### 5.4 Yellow Emission

Te^4+^ ions belong to the ns^2^ group and are widely used as sensitizers in double perovskites or as dopant ions to induce a yellow emission in a host material.^29^ Despite the size and charge differences, Te^4+^ ions are able to replace Zn^2+^ ions in tetrahedral sites to create [TeCl_4_] clusters, which acts as a luminescent center from which the yellow emission emerges via an extrinsic STE emission mechanism (Figure 16a,b).^48^ Upon successful doping, the PLE spectrum of Te^4+^-doped Cs_2_ZnCl_4_ shows two bands in the 250–500 nm range, as seen also in Cs_2_TeCl_6_. The long-wavelength excitation (A band) is attributed to ^1^S_0_ → ^3^P_1_ transitions, while the short-wavelength excitation (B and C bands) are ascribed to the ^1^S_0_ → ^3^P_0_ and ^1^S_0_ → ^3^P_2_ transitions of Te^4+^ ions (Figure 16c).^48^

**Figure 16:**
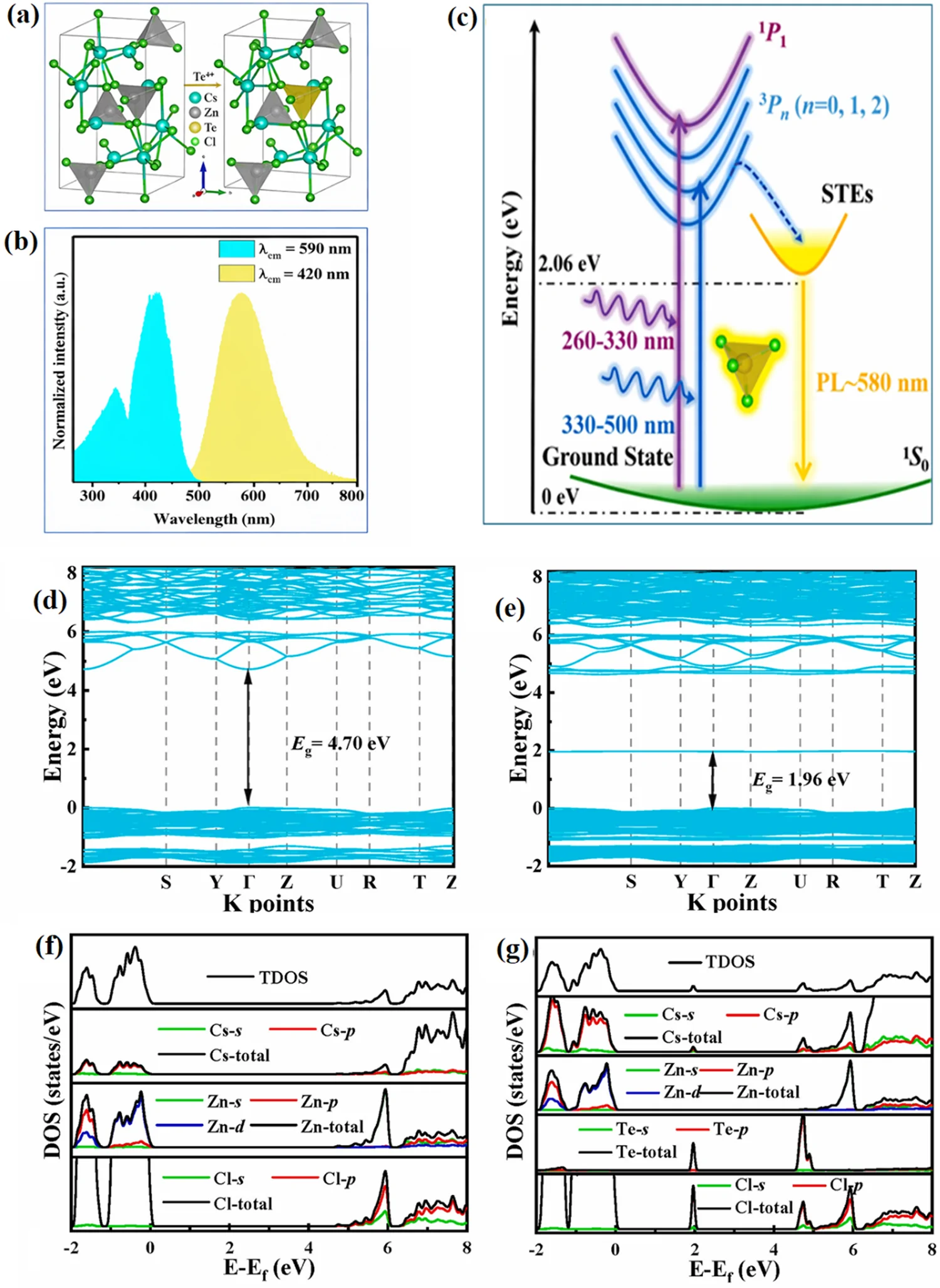
(a) Te^4+^ ions replacing Zn^2+^ ions. (b) PL and PLE spectra of Te^4+^-doped Cs_2_ZnCl_4_. (c) Electronic transition and energy splitting in Te^4+^-doped Cs_2_ZnCl_4_. (d) Band structure of Cs_2_ZnCl_4_. (e) Band structure of Te^4+^-doped Cs_2_ZnCl_4_. (f) PDOS of Cs_2_ZnCl_4_. (g) PDOS of Te^4+^-doped Cs_2_ZnCl_4_. Reproduced from ref 48. Copyright 2025, Elsevier.

Doping Zn halides with Te^4+^ ions is a good strategy to overcome the limitations imposed by the indirect bandgap and low PLQY in Cs_2_TeCl_6_ double perovskites.^33,70^ The reason is that Zn metal halides host exhibits a direct bandgap, while Cs_2_TeCl_6_ double perovskites have an indirect bandgap, which contributes to its extremely low PLQY values as low as 2.06%.^33,70^ Upon successful doping of Te^4+^ ions into Cs_2_ZnCl_4_, a broadband yellow emission centered at 590 nm is observed, similar to that of pristine Cs_2_TeCl_6_, with a PLQY of 33.7% and a FWHM of 117 nm.^48^ As for the exciton recombination dynamics, TRPL measurements reveal that Te^4+^-doped Cs_2_ZnCl_4_ has an average radiative decay lifetime of 52.14 ns, which is close to the radiative decay lifetime in Cs_3_TeCl_6_ double perovskites that shows an average radiative decay lifetime of 71.9 ns.^71^ Upon Te^4+^ doping, an impurity band or midgap state emerges at the CBM. This leads to a significant reduction in the bandgap, from 4.7 to 1.96 eV, while still maintaining a direct bandgap (Figure 16d–g) and Table 7.^48^

**Table 7:** Band Structure and DOS of Cs_2_ZnCl_4_ and Te^4+^-Doped Cs_2_ZnCl_4_

material	band gap (eV)	band gap type	(VBM)	(CBM)	refs
Pristine Cs_2_ZnCl_4_	4.7	Direct (Γ point)	Cl-3p, Zn-3d	Zn-4s, Cl-3p	^48^
Te^4+^-doped Cs_2_ZnCl_4_	1.96 (midgap state)	Direct (Γ point)	Te-5s, Cl-3p	Te-5p, Cl-3p	^48^

### 5.5 Red-NIR Emission

Despite the tunable emission in 0D metal halides, it must be emphasized that near-infrared (NIR) emission is rare in these materials. Most 0D metal halides show PL emission within the visible range. However, their PL emission can be extended to the NIR range using dopant ions such as Sn^2+^, Sb^3+^, Mo^4+^, W^4+^, Cr^3+^, and trivalent lanthanide (Ln^3+^) ions. Transition metal ions, such as Mo^4+^, W^4+^, Cr^3+^, show broadband NIR emission, whereas Ln^3+^ ions show narrow PL emission.^5,34^

#### 5.5.1 Sb^3+^-Doped Zn Metal Halides

Sb^3+^ ions are widely used as dopants to induce new luminescent centers or employed as sensitizers to enhance absorption and emission intensities of a host material.^56^ As discussed in previous sections, Cs_2_ZnCl_4_ exhibits a weak broadband emission centered at 512 nm at room temperature, with a PLE maximum at 267 nm.^56^ However, its doped counterpart, Sb^3+^-doped Cs_2_ZnCl_4_, shows two excitation peaks located at 266 and 316 nm. The peak at 266 nm is attributed to the Cs_2_ZnCl_4_ host, while the peak at 316 nm is ascribed to disphenoidal [SbCl_4_]^−^ polyhedra (Figure 17a,b).^56^

**Figure 17:**
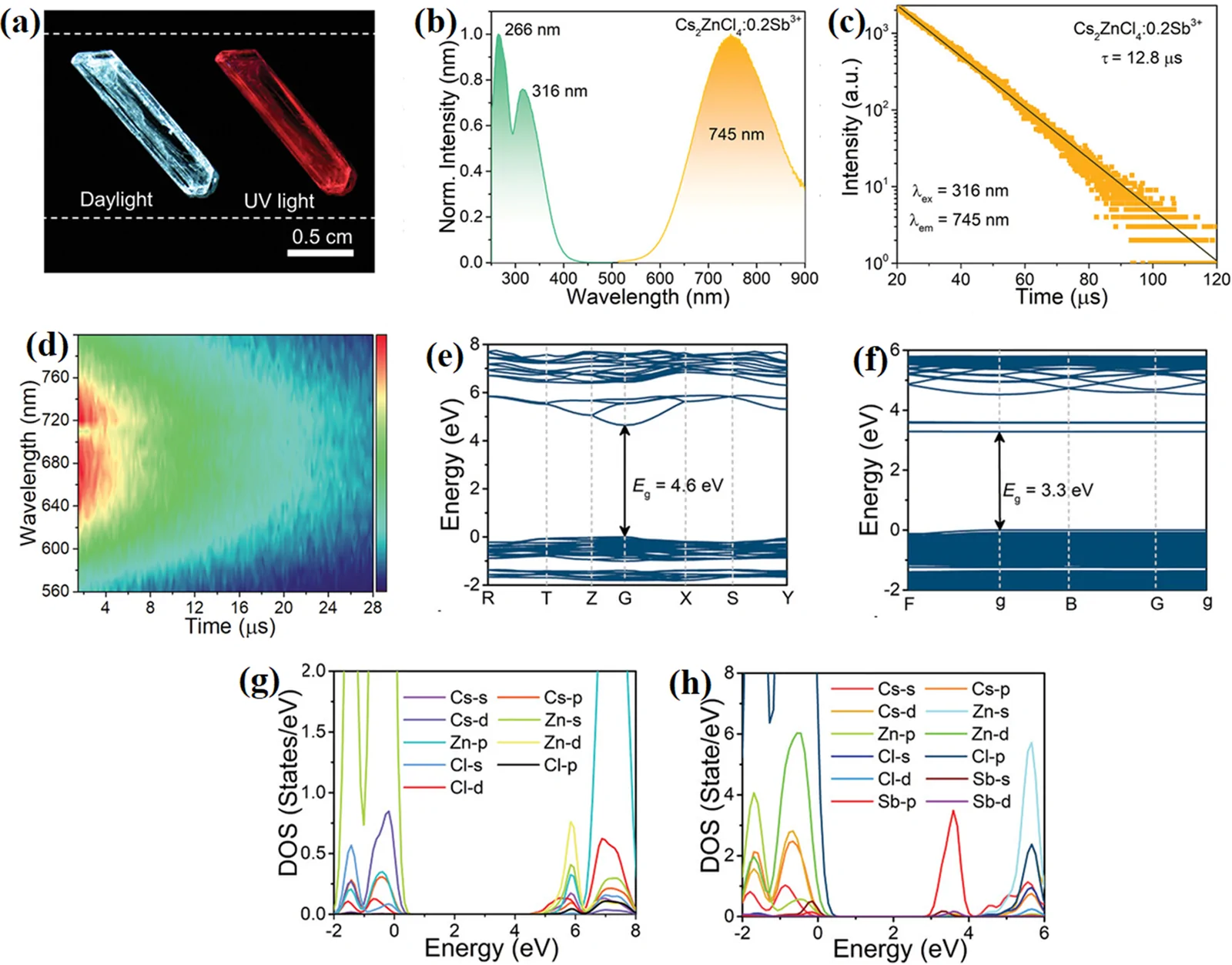
(a) Sb^3+^-doped Cs_2_ZnCl_4_ under UV light. (b) PL and PLE spectra of Sb^3+^-doped Cs_2_ZnCl_4_. (c) TRPL spectra of Sb^3+^-doped Cs_2_ZnCl_4_. (d) Pseudocolor map of transient emission spectra. (e) Band structure of Cs_2_ZnCl_4_. (f) Band structure of Sb^3+^-doped Cs_2_ZnCl_4_. (g) DOS of Cs_2_ZnCl_4_. (h) DOS of Sb^3+^-doped Cs_2_ZnCl_4_. Reproduced from ref 56. Copyright 2021, Wiley.

Upon excitation at 316 nm, Sb^3+^-doped Cs_2_ZnCl_4_ revealed a broadband (NIR) emission peak centered at 745 nm, attributed to triplet STE emission disphenoidal [SbCl_4_]^−^ polyhedra. As expected, the long microsecond radiative decay lifetime (12.8 μs) indicates that the NIR emission originates via STE emission in [SbCl_4_]^-^ units (Figure 17c,d). In addition, Sb^3+^ doping also reduced the bandgap, introduced an additional absorption band, and made the band edges more localized (Figure 17e–h). In Cs_2_ZnCl_4_, the VBM is contributed by Zn-3d and Cl-3p, while the CBM is made of Zn-4s and Cl-3p. As for Sb^3+^-doped Cs_2_ZnCl_4_, its VBM is composed of Sb-5s and Cl-3p, while the CBM is made of Sb-5p and Cl-3p (Table 8).

**Table 8:** Band Structure and DOS of Cs_2_ZnCl_4_ and Sb^3+^-Doped Cs_2_ZnCl_4_

material	band gap (eV)	band gap type	(VBM)	(CBM)	refs
Pristine Cs_2_ZnCl_4_	4.6	Direct (Γ point)	Zn-3d, Cl-3p	Zn-4s, Cl-3p	^56^
Sb^3+^-doped Cs_2_ZnCl_4_	3.3	Direct (Γ point)	Sb-5s, Cl-3p	Sb-5p, Cl-3p	^56^

#### 5.5.2 Sn^2+^-Doped Zn Metal Halides

Divalent Sn^2+^ ions can induce a red-NIR emission in Zn metal halides by substituting Zn^2+^ ions at tetrahedral sites. Figure 18a demonstrates that the absorption intensity increases with increasing Sn^2+^ dopant concentration up to 2.5%.^40^ Upon excitation at 320 nm, Sn^2+^-doped Cs_2_ZnBr_4_ shows a broadband NIR emission centered at 698 nm with a large Stokes shift of 323 nm, modest PLQY of 41%, and a radiative decay lifetime of 7.81 μs (Figure 18b,c).^40^ Most interestingly, temperature-dependent PL spectra measured in the range of 77–227 K revealed that the NIR PL emission increased as the temperature was lowered to 77K due to suppressed lattice vibrations and thermal-induced nonradiative recombination (Figure 18d).^40^ Moving on, the calculated Cs_2_ZnBr_4_ exhibits a direct bandgap of 3.742 eV, with the VBM mainly from Br-4p and Zn-3d orbitals, and the CBM is from Br-4p and Zn-4s orbitals. In contrast, Sn^2+^-doped Cs_2_ZnBr_4_ shows a reduced bandgap of 2.939 eV, where VBM is dominated by Zn-3d, Sn-5s, and Br-4p orbitals, and CBM is composed of Sn-5p and Br-4p orbitals (Figure 18e–h and Table 9).^40^

**Figure 18:**
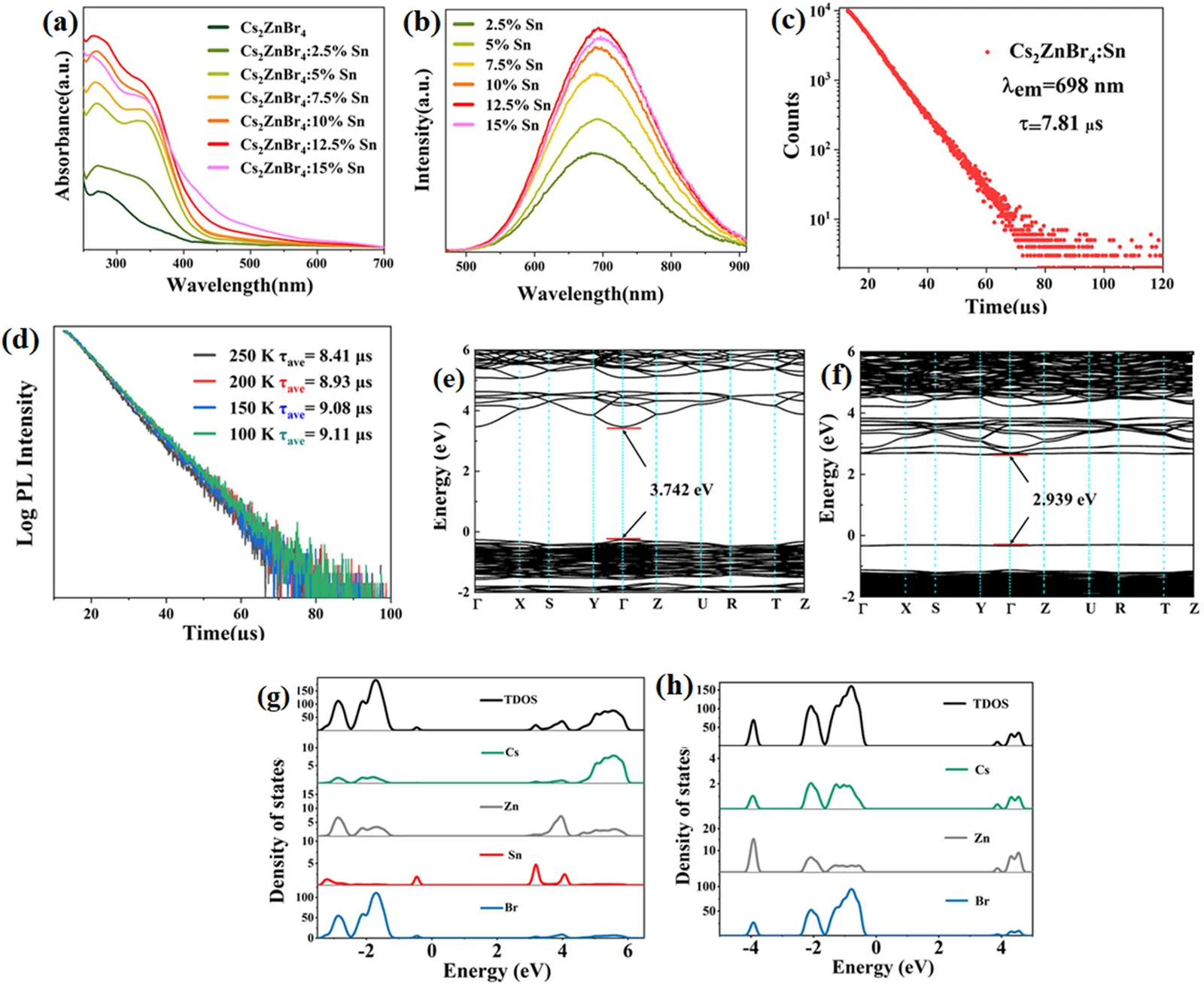
(a) PLE spectra of Sn^2+^-doped Cs_2_ZnBr_4_. (b) PL spectra of Sn^2+^-doped Cs_2_ZnBr_4_. (c) TRPL of Sn^2+^-doped Cs_2_ZnBr_4_. (d) TRPL at different temperatures. (e) Band structure of Cs_2_ZnBr_4_. (f) Band structure of Sn^2+^-doped Cs_2_ZnBr_4_. (g) DOS of Cs_2_ZnBr_4_. (h) DOS of Sn^2+^-doped Cs_2_ZnBr_4_. Reproduced from ref 40. Copyright 2023, Wiley.

**Table 9:** Band Structure and DOS of Cs_2_ZnBr_4_ and Sn^2+^-Doped Cs_2_ZnBr_4_ ^40^

material	band gap (eV)	band gap type	(VBM)	(CBM)	refs
Pristine Cs_2_ZnBr_4_	3.742	Direct (Γ point)	Zn-3d, Br-4p	Zn-4s, Br-4p	^40^
Sn^2+^-doped Cs_2_ZnBr_4_	2.939	Direct (Γ point)	Sn-5s, Br-4p	Sn-5p, Br-4p	^40^

#### 5.5.3 Cr^3+^ Doped Zn Metal Halides

Cr^3+^ ions are widely employed to induce infrared emission that span from 800 nm to approximately 1300 nm, depending on the host material. The near-infrared emission originates from the d-d electronic transitions of the Cr^3+^ ions.^72^ In the context of Zn metal halides, upon incorporation of Cr^3+^ into the lattice, the Cr^3+^ ions replace Zn^2+^ ions at tetrahedral sites. In Figure 19a,b, the diffraction peaks are shifted to higher angles with increasing Cr^3+^ concentration, reflecting lattice contraction due to the smaller ionic radius of Cr^3+^ (0.46 Å, CN = 4) compared to Zn^2+^ (0.6 Å, CN = 4), which confirms the successful substitution of Zn^2+^ with dopant Cr^3+^ at tetrahedral sites.^72^ Based on defect chemistry, the charge difference between Zn^2+^ and Cr^3+^ introduces a positive charge imbalance. Therefore, this positive charge imbalance is compensated by the formation of anion vacancies to maintain charge neutrality.^72^ The PL and PLE spectra in Figure 19c,d show a broadband NIR emission centered at 915 nm due to ^4^T_2_ (^4^F) → ^4^A_2_ (F) transitions with a PLQY ∼ 16.91%. The PLE spectra reveal three excitation peaks ^4^A_2_ (F) → ^4^T_1_ (^4^P) at 375 nm, ^4^A_2_ (F) → ^4^T_1_ (^4^F) at 532 nm, and ^4^A_2_ (F) → ^4^T_2_ (^4^F) at 735 nm.^72,73^ These are attributed to Cr^3+^ related d-d transitions. From these results, it is anticipated that Zn metal halides should be able to accommodate other NIR-emitting transition metals, such as Ni^2+^, Mo^4+^, and W^4+^ ions.

**Figure 19:**
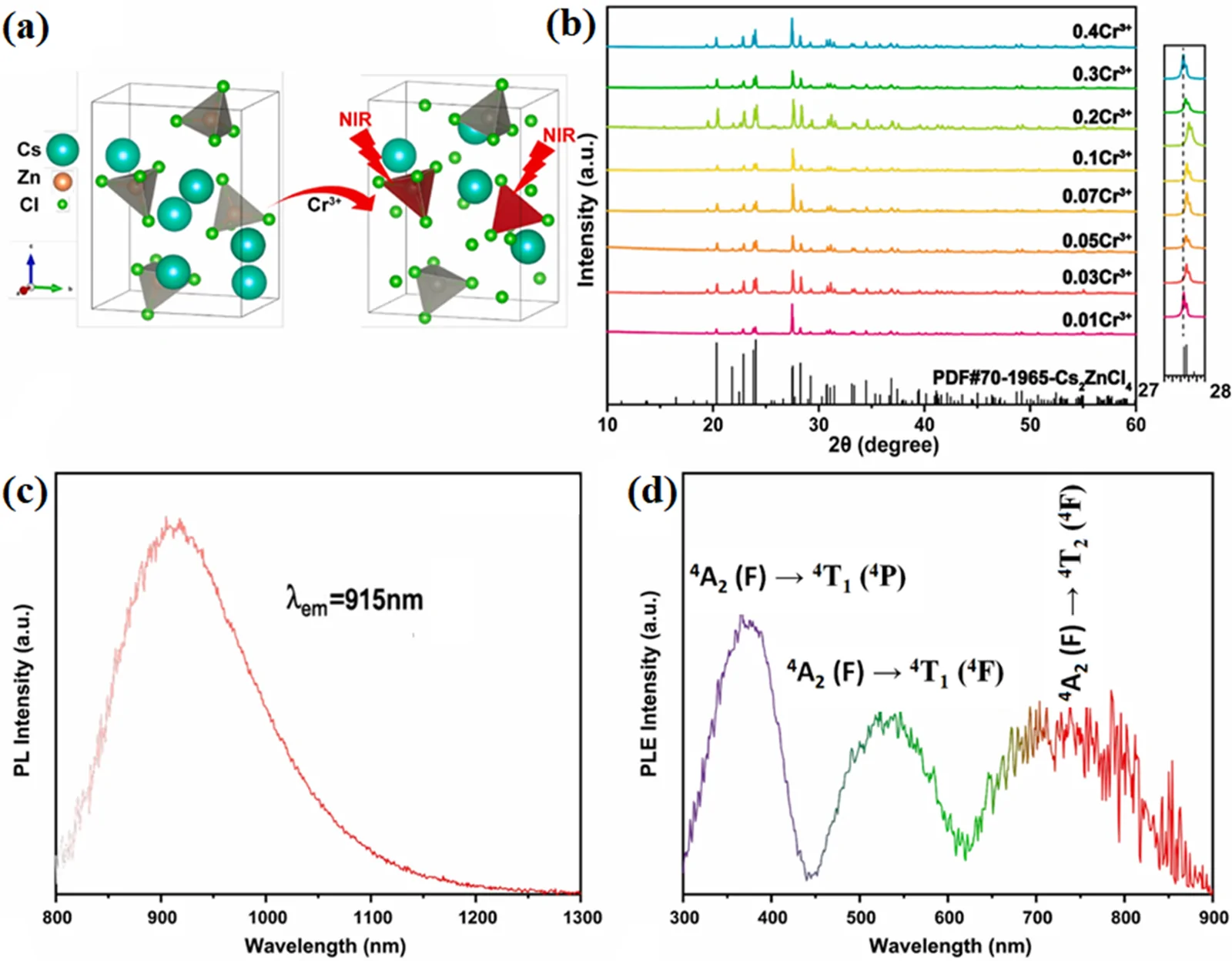
(a) Incorporation of Cr^3+^ into Cs_2_ZnCl_4_. (b) Lattice contraction due to Cr^3+^ incorporation. (c) PL spectra of Cr^3+^-doped Cs_2_ZnCl_4_. (d) PLE spectra of Cr^3+^-doped Cs_2_ZnCl_4_. Reproduced from ref 72. Copyright 2024, Elsevier.

## 6 Optoelectronics Applications

Thus far, 0D Zn metal halides have been demonstrated to be a low-cost and solution-processable semiconductor that is highly suitable for optoelectronic applications. The PL emission and absorption wavelengths in 0D Zn metal halides can be tuned using dopant ions. This section discusses the potential of 0D materials for optoelectronic applications, namely X-ray scintillation, solid-state phosphors, and anticounterfeiting.

### 6.1 X-ray Scintillation

At present, there is a high demand for flexible, low-cost X-ray scintillating materials for medical imaging and security inspection.^74^ In general, a scintillator is a material that converts high-energy X-ray photons into low-energy visible light photons (Figure 20a,b).^74,75^ Intensive research is underway, with numerous reports demonstrating the use of metal halides such as CsPbBr_3_, Cs_3_Cu_2_I_5_, Cs_2_ZrCl_6_, Cs_2_HfCl_6_, and Cs_3_CeCl_6_ as scintillators.^5^ 0D metal halides are competent candidates as scintillators due to their high PLQY, intense X-ray absorption coefficient, high spatial resolution, phenomenal light yield and large Stokes shift.^74^ Moreover, 0D metal halides like Cs_3_Cu_2_I_5_ and Cs_3_TbCl_6_ outperform some conventional commercialized inorganic scintillators with respect to X-ray absorption coefficient, light yield, and detection limit. Based on the results presented in (Figure 20c–f), Zn metal halides should show similar scintillation performance as their 0D metal halide counterparts.^76,77^

**Figure 20:**
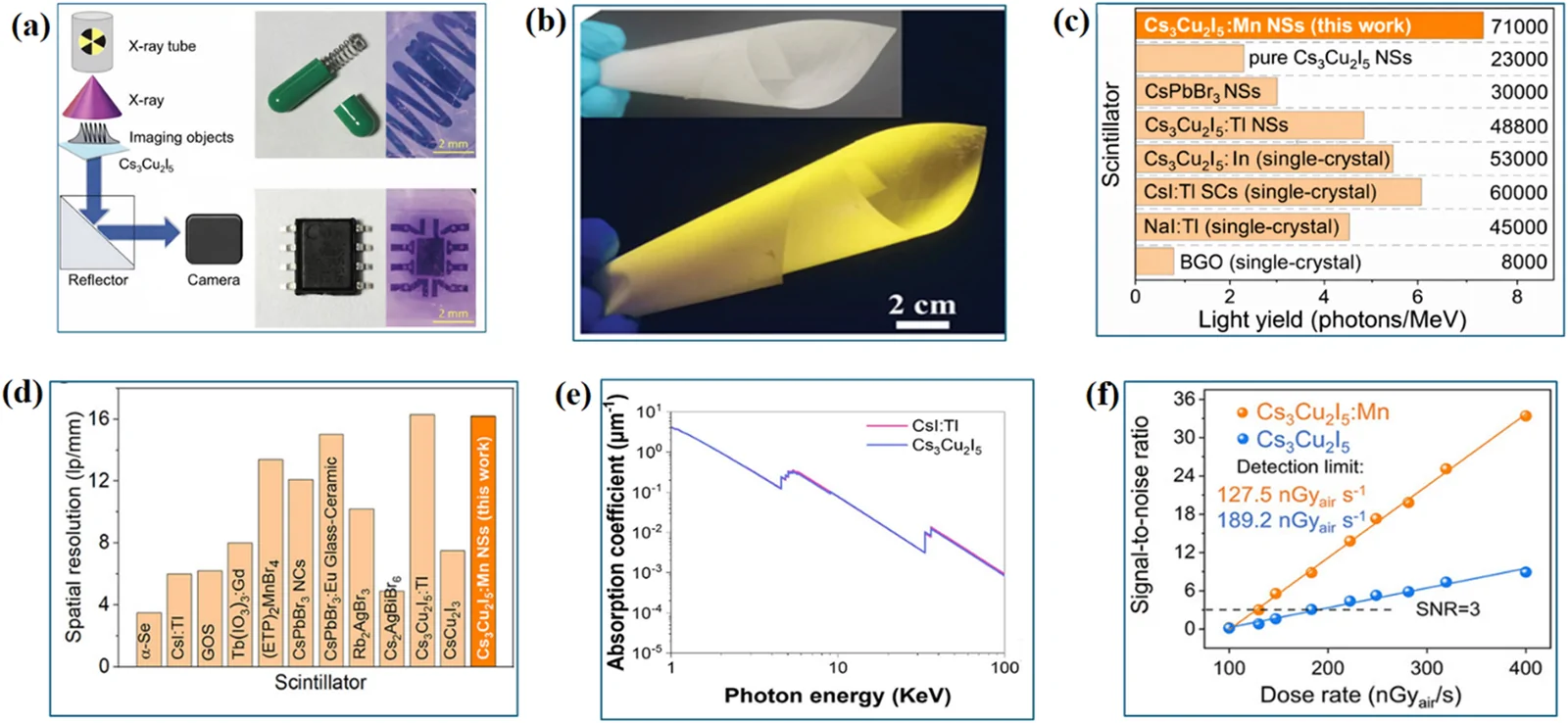
(a) X-ray scintillation setup. Reproduced from ref 74. Available under a CC-BY 4.0 license. Copyright 2022, Wiley. (b) Flexible PMMA film of CsCu_2_I_3_. Reproduced from ref 75. Copyright 2023, Wiley. (c) Light yield of Cs_3_Cu_2_I_5_. (d) Spatial resolution of Cs_3_Cu_2_I_5_. (e) Cs_3_Cu_2_I_5_ vs Cs:Tl absorption coefficient. Reproduced from ref 76. Copyright 2023, American Chemical Society. (f) Signal-to-noise ratio vs dose rate. Reproduced from ref 77. Copyright 2024, Wiley.

### 6.2 Solid-State Phosphors

Phosphors are solid materials that absorb light at a certain wavelength and emit it at another wavelength. Zn metal halides can be used as a potential alternative to conventional commercial silicate and nitrate phosphors. This subsection discusses the potential applications of these materials as luminescent phosphors for solid-state lighting, owing to their tunable or modular emission properties.

#### 6.2.1 UV-Emitting Phosphors

One of the most common methods to achieve strong UV emission is through doping Ce^3+^ in silicate or nitrate hosts. Likewise, UV emission can also be induced in Zn metal halides using Ce^3+^ dopants. As a matter of fact, Ce^3+^-doped Zn metal halides show phenomenal anti-thermal PL quenching resistance, which is rare and not found in most host materials (Figure 21a).^1^ Furthermore, Ce^3+^-doped Cs_2_ZnBr_4_ also demonstrates an impressive T_50_ operational time under 20, 40, and 60 mA (Figure 21b,c).^1^ These results represent some of the best anti-thermal PL quenching resistance reported for 0D metal halides. Figure 21d illustrates the antithermal quenching mechanism, in which the Ce^3+^ 5d orbitals constitute the conduction band, while the Ce^3+^ 4f orbitals contribute to the valence band.^1^ At the same time, it must be kept in mind that heterovalent doping with Ce^3+^ ions causes substitutional defects and local positive centers near the 5d state that act as electron traps.^1^ These traps can be seen as a storage site or a reservoir into which a portion of the excited electrons get trapped. At high temperatures, nonradiative recombination is expected to dominate, which would normally reduce PL emission.^1^ However, at high temperatures, electrons trapped in defect levels overcome the trap potential and recombine radiatively to compensate for nonradiative pathways.^1^

**Figure 21:**
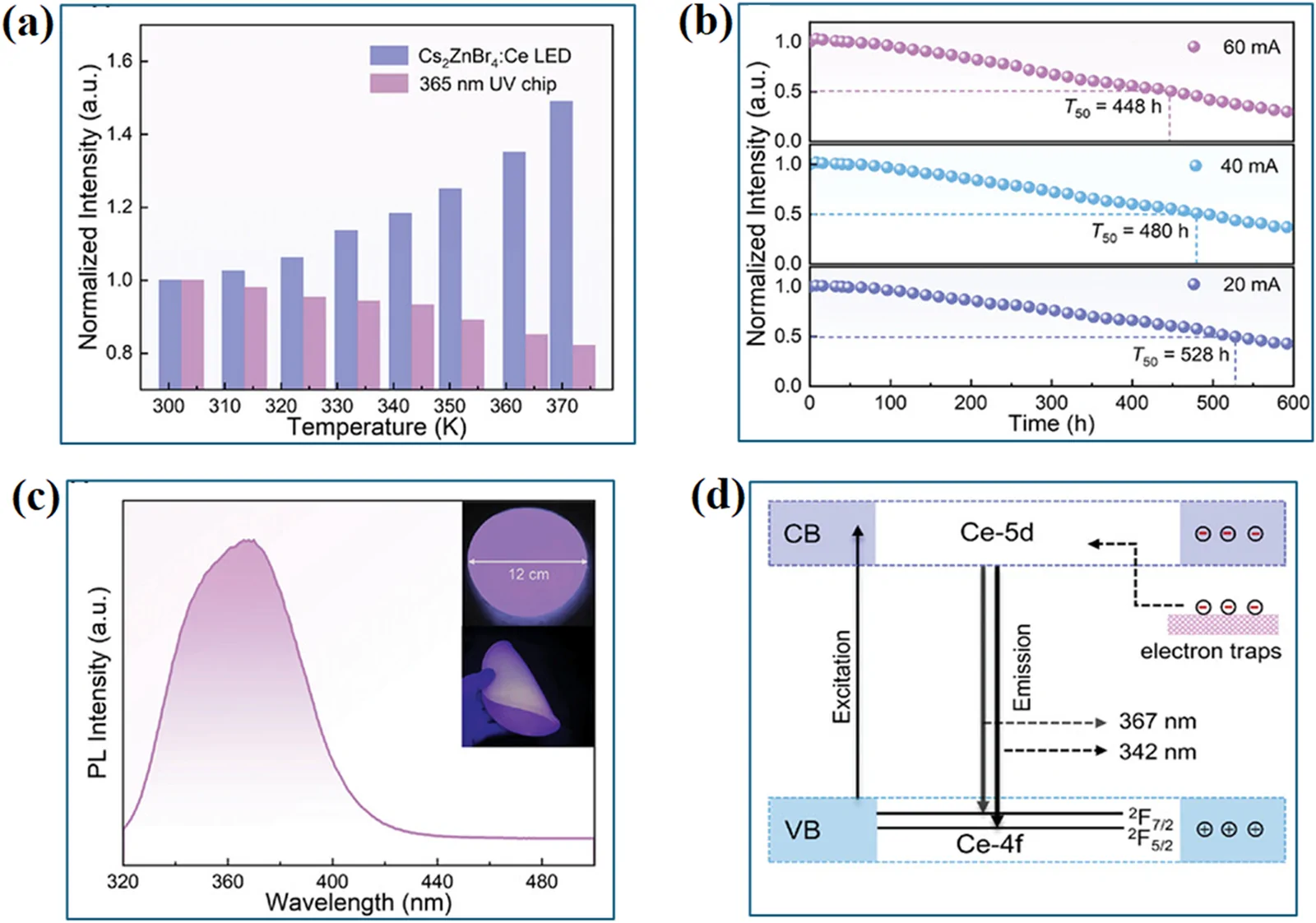
(a) Normalized intensity of UV-pumped Ce^3+^-doped Cs_2_ZnBr_4_ between 300 and 370 K. (b) Operational stability of Ce^3+^-doped Cs_2_ZnBr_4_ at different drive currents. (c) PL spectra of Ce^3+^-doped Cs_2_ZnBr_4_. (d) Anti-thermal PL quenching mechanism of Ce^3+^-doped Cs_2_ZnBr_4_. Reproduced from ref 1. Copyright 2025, Wiley.

#### 6.2.2 Luminescent Phosphors

Zn metal halides are good host materials that can be doped to achieve a desired emission wavelength spanning the UV to the NIR regions of the electromagnetic spectrum. Monochromatic phosphors that emit a single color can be obtained using single dopant ions. For example, Cu^+^-doped Cs_2_ZnBr_4_ shows blue emission, while Mn^2+^-doped Cs_2_ZnBr_4_ exhibits green emission.^3^ Multichromatic applications, such as white LEDs, require multiple emissions centers. Dopant ions, such as Bi^3+^, Mn^2+^, and Eu^3+^, can be doped into the lattice, and upon excitation at 365 nm, a bright white emission with a high color rendering index (CRI) can be achieved.

#### 6.2.3 NIR Emission Phosphors

NIR emissions can be induced in Zn metal halides using dopants such as Sn^2+^, Sb^3+^, and Cr^3+^ ions.^40,56,72^ These ions are known to adopt octahedral coordination in ABX_3_ or A_2_BX_6_ metal halides. However, reports on Zn metal halides indicate that these ions can also adopt a tetrahedral coordination geometry. Clarifying the coordination geometry is critical because a tetrahedral environment can negate the Laporte-forbidden selection rule in d-block transition metals such as Mo^4+^, Cr^3+^, and W^4+^, and thereby improving absorption and emission intensities.^5^ As shown in Figure 22a, Sb^3+^-doped Cs_2_ZnCl_4_ was integrated onto a 365 nm LED chip to generate an intense NIR emission peak centered at 745 nm, with a FWHM of 175 nm, a Stokes shift of 429 nm, and a high PLQY of 69.9%.^56^ The NIR emission increased as the drive current increased from 20 mA to 300 mA (Figure 22b,c). Most importantly, Sb^3+^-doped exhibits good performance for night vision applications (Figure 22d,e). Likewise, Sn^2+^-doped Cs_2_ZnBr_4_ showed a similar broadband NIR emission material at 700 nm, with a high PLQY of 41% and a FWHM of 177 nm.^56^

**Figure 22:**
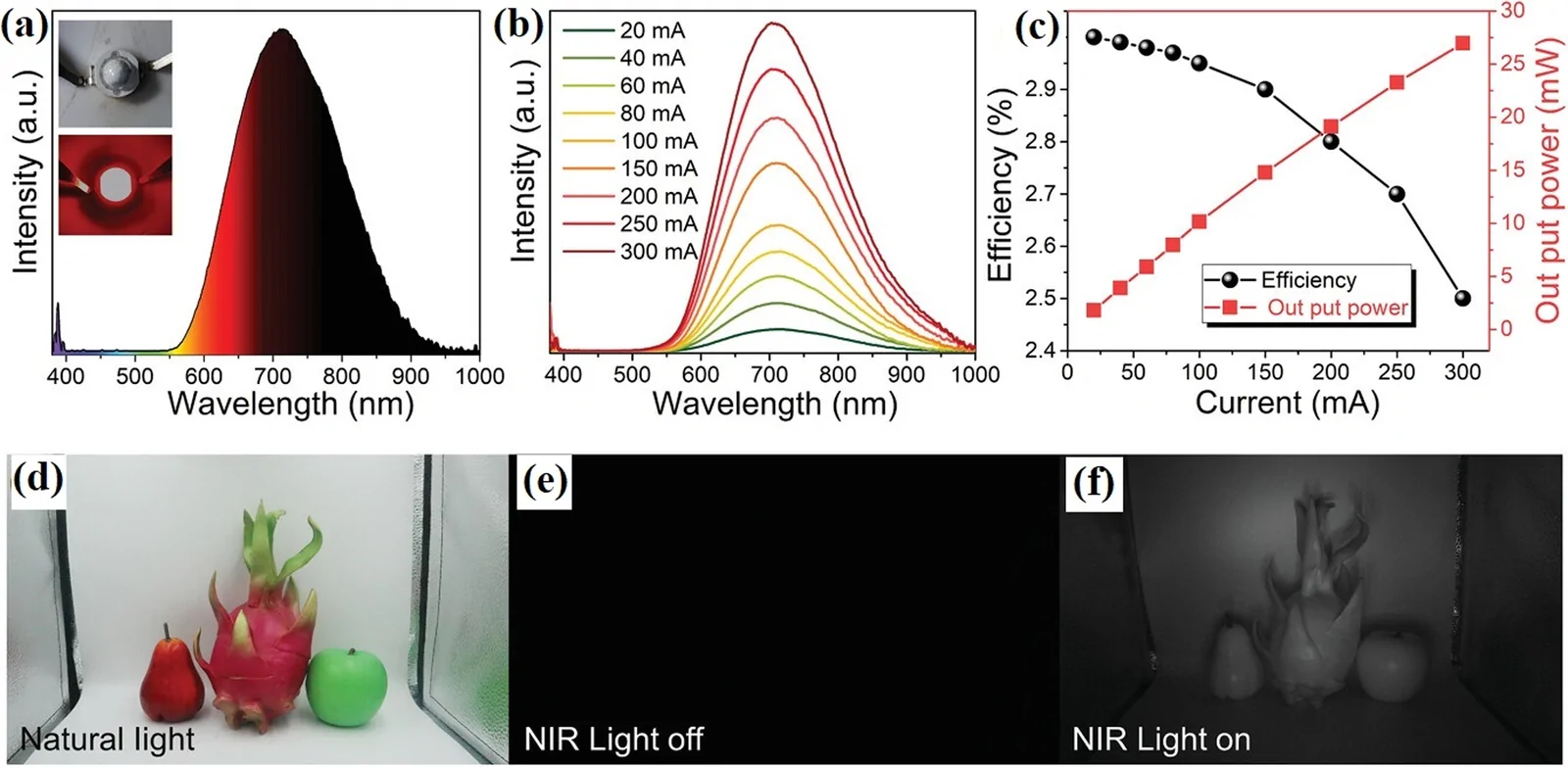
(a) Emission spectrum of Sb^3+^-doped Cs_2_ZnCl_4_. (b) Driven current-dependent emission spectra of Sb^3+^-doped Cs_2_ZnCl_4_. (c) Variation of near-infrared (NIR) emission power and photoelectric conversion efficiency as a function of applied forward current. (d) Photograph obtained under ambient lighting using a standard visible camera. Images captured by NIR camera under NIR LED light (e) Switched off and (f) Switched on. Reproduced from ref 56. Copyright 2021, Wiley.

### 6.3 Anticounterfeiting

Zn metal halides are promising anticounterfeiting materials because they can host multiple dopant ions with different excitation wavelengths.^78^ This means that the emission wavelength can be modulated by selecting dopant ions that are excited at different wavelengths. Taking Cu^+^/Mn^2+^ codoped Cs_2_ZnBr_4_ as an example, when excited at 254 nm, one sees 8888 code with blue emission, whereas when excited at 365 nm, one observes 0407 code with green emission.^78^ The Cu^+^ dopants are excited at 254 nm, whereas Mn^2+^ is excited at 365 nm (Figure 23).^78^ Therefore, this excitation-dependent emission can be regarded as a unique optical signature that makes Zn metal halides strong candidates for anticounterfeiting applications because such excitation-dependent emission behavior is extremely difficult to replicate.

**Figure 23:**
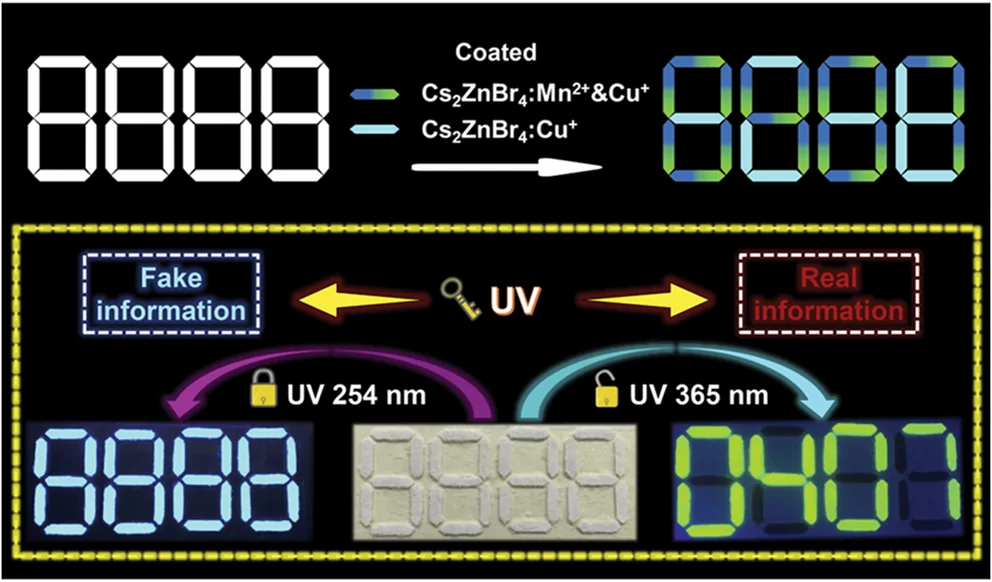
Anticounterfeiting behavior in Cu^+^ and Mn^2+^ codoped Cs_2_ZnBr_4_ under 254 and 365 nm UV light. Reproduced from ref 78. Copyright 2022, IOP Publishing.

## 7 Conclusion and Future Outlook

Zinc metal halides show optical properties that are quite unique in comparison to other metal halides, such as CsPbX_3_, Cs_3_Cu_2_X_5_, or Cs_3_MnX_5_. Throughout this review, we have demonstrated Zn metal halides as host materials that can be doped with luminescent dopant ions to achieve a desired emission wavelengths. In general, Zn metal halides are divided into Cs_2_ZnX_4_ and Cs_3_ZnX_5_ with a tetrahedral crystal structure. The most interesting aspect with regard to Zn metal halides is that these materials are low-cost and solution-processable. The synthesis temperature can range from room temperature to 180 °C. Zn metal halides exhibit a soft ionic lattice with strong electron–phonon coupling, similar to other metal halides such as Cs_3_Cu_2_X_5._ As a result, when dopant ions are introduced into the lattice, they replace Zn^2+^ ions at the tetrahedral sites to form metal halide clusters, resulting in dopant-induced STE emissions. Moreover, doped Zn metal halides demonstrate good potential as optoelectronic materials owing to high absorption coefficients, high defect tolerance, large Stokes shift, and a high PLQY. Nonetheless, despite these interesting properties, several challenges remain to be addressed and uncharted research directions remain to be explored in order to harness the potential of Zn metal halides. For instance, thermal quenching is a significant challenge that needs to be solved. The reason is that the emission intensity in most metal halides decreases at high temperatures due to thermal-induced PL quenching. However, theoretical calculations can be conducted to investigate which dopant ions can induce shallow defects, which may lead to anti-thermal PL quenching behavior. In simple words, thermal-induced PL quenching can be inhibited by defect engineering, which helps maintain luminescence at high temperatures. Although most reports have stated that Cr^3+^, W^4+^, or Mo^4+^ adopt a tetrahedral coordination geometry in Zn metal halides. However, further investigation is needed to confirm this coordination geometry. The reason is that tetrahedral geometry is not restricted to the Laporte-forbidden selection rules, which is the root cause of the low absorption and emission intensities in transition metals with NIR emission via d-d transitions in octahedral geometry. Therefore, it is plausible that the NIR emission in doped Zn metal halides should be stronger compared to the NIR emission in octahedral coordinated structures like ABX_3_ or A_2_BX_6_. Likewise, trivalent Ln^3+^ dopants should be explored. Each Ln^3+^ ion has a unique emission wavelength, which can expand the applications of Zn metal halide hosts in niche areas, such as solid-state lighting, display technology and optical communication. Another interesting research direction is the fabrication of large-area scintillators. Currently, most X-ray scintillation applications of metal halides are demonstrated using small-area films, which are not suitable for real-world applications. Future research should head toward large-area X-ray scintillators, which are more practical. The synthesis should be scaled up, and the film area should be larger. Overall, this article should contribute to the understanding of the impact of dopant ions in inducing new luminescent centers and modulating the optical and electronic properties of Zn metal halides.
